# HetF Protein Is a New Divisome Component in a Filamentous and Developmental Cyanobacterium

**DOI:** 10.1128/mBio.01382-21

**Published:** 2021-07-13

**Authors:** Wei-Yue Xing, Jing Liu, Zi-Qian Wang, Ju-Yuan Zhang, Xiaoli Zeng, Yiling Yang, Cheng-Cai Zhang

**Affiliations:** a State Key Laboratory of Freshwater Ecology and Biotechnology, Chinese Academy of Sciences, Wuhan, China; b Key Laboratory of Algal Biology, Institute of Hydrobiology, Chinese Academy of Sciences, Wuhan, China; c University of Chinese Academy of Sciences, Beijing, China; d Institute WUT-AMU, Aix-Marseille University and Wuhan University of Technology, Wuhan, China; e Innovation Academy for Seed Design, Chinese Academy of Sciences, Beijing, China; Pasteur Institute

**Keywords:** cell division, FtsZ, heterocysts, cyanobacteria, peptidoglycan

## Abstract

Bacterial cell division, with a few exceptions, is driven by FtsZ through a treadmilling mechanism to remodel and constrict the rigid peptidoglycan (PG) layer. Yet different organisms may differ in the composition of the cell division complex (divisome). In the filamentous cyanobacterium *Anabaena* sp. strain PCC 7120, *hetF* is required for the initiation of the differentiation of heterocysts, cells specialized in N_2_ fixation under combined-nitrogen deprivation. In this study, we demonstrate that *hetF* is expressed in vegetative cells and necessary for cell division under certain conditions. Under nonpermissive conditions, cells of a Δ*hetF* mutant stop dividing, consistent with increased levels of HetF under similar conditions in the wild type. Furthermore, HetF is a membrane protein located at midcell and cell-cell junctions. In the absence of HetF, FtsZ rings are still present in the elongated cells; however, PG remodeling is abolished. This phenotype is similar to that observed with the inhibition of the septal PG synthase FtsI. We further reveal that HetF is recruited to or stabilized at the divisome by interacting with FtsI and that this interaction is necessary for HetF function in cell division. Our results indicate that HetF is a member of the divisome depending mainly on light intensity and reveal distinct features of the cell division machinery in cyanobacteria that are of high ecological and environmental importance.

## INTRODUCTION

Bacterial cell division is a highly complex and coordinated event that has been extensively studied in model bacteria (1, 2). In Escherichia coli, cell division starts with the polymerization of a tubulin-like protein, FtsZ, at midcell, forming a membrane-associated structure called a Z-ring that is stabilized and anchored to the inner cell membrane by proteins such as FtsA and ZipA (3, 4). This early Z-ring complex then serves as a scaffold for the sequential recruitment of other cell division proteins, such as FtsK, FtsQ, FtsL, FtsB, FtsW, and FtsN, that are involved in chromosome segregation, the structural integrity of the protein complex, or the transfer of precursors required for cell wall synthesis (5, 6). Next, more cell division proteins related to peptidoglycan (PG) synthesis (such as FtsI, penicillin binding protein 1b [PBP1b], and LpoB) or hydrolysis (such as AmiA, AmiB, and AmiC) are assembled into the structure, forming a large cell division complex called the divisome, which further executes septum formation (6). When cell constriction starts, FtsZ guides PG remodeling at the outer surface of the plasma membrane through a treadmilling mechanism (7), which leads to the separation of the two daughter cells.

Although many components of the divisome are conserved in bacteria, some proteins of the machinery diverge significantly in subgroups of bacteria (8–10). In cyanobacteria, FtsZ is also conserved, as are many proteins involved in the structural stability of the divisome and cell wall synthesis or chromosome segregation, such as FtsI (PBP3), PBP1b, AmiA, AmiB, AmiC, FtsK, FtsQ, and FtsW. Other components, identified in cyanobacteria but not found in E. coli, are either shared by Gram-positive bacteria such as Bacillus subtilis or specific to cyanobacteria. This is the case, for example, for ZipN (Ftn2), ZipS (Ftn6), and SepF (Cdv2) in cyanobacteria (11–15), which are functionally equivalent to FtsA and ZipA in E. coli. The composition of the cyanobacterial divisome has been investigated, and a network of interactions was revealed among many of these cell division-related proteins (11–17). Despite all the effort, the cell division mechanism in cyanobacteria is still poorly understood in comparison to those of model organisms such as E. coli, B. subtilis, Staphylococcus aureus, and Streptococcus pneumoniae (6, 18–22).

As Gram-negative prokaryotes, cyanobacteria are ubiquitous and play essential roles in element cycles. Some cyanobacteria are able to differentiate into different cell types for the division of labor. This is the case for *Anabaena* sp. strain PCC 7120 (here, *Anabaena*), which is able to perform oxygen-labile N_2_ fixation using heterocysts, a specialized cell type regularly intercalated among vegetative cells that perform oxygen-evolving photosynthesis (23, 24). Heterocysts account for 5 to 10% of the total cells on the filaments and are induced upon the deprivation of combined nitrogen in the growth medium. Heterocyst differentiation takes about 20 to 24 h, during which extensive morphological and physiological changes occur (23–25). Heterocyst development involves a complex regulatory network, among which NtcA and HetR are the central factors (26–29). HetF is another regulator that has been shown to be essential for heterocyst development in both Nostoc punctiforme ATCC 29133 and *Anabaena* (30, 31). HetF is a putative protease, and mutation of the conserved active site led to a loss-of-function phenotype. Interestingly, beyond its function in cell differentiation, one report showed that a *hetF* mutant displays morphological changes such as cell elongation and/or a larger cell size (30), suggesting that *hetF* may also play a role in cell division. However, the occurrence of such morphological changes was not consistently observed in the published data or even in the same study (30–33). Therefore, the function of *hetF* in cell division needs to be clarified.

Based on different approaches, we show here that HetF is involved in septal PG synthesis as a member of the divisome under conditions of increasing light intensity, especially when nitrate is used as the nitrogen source. These results advance our understanding of the cell division mechanism in cyanobacteria that are of ecological and environmental importance.

## RESULTS

### *hetF* is expressed in vegetative cells and downregulated in mature heterocysts.

**FIG 1:**
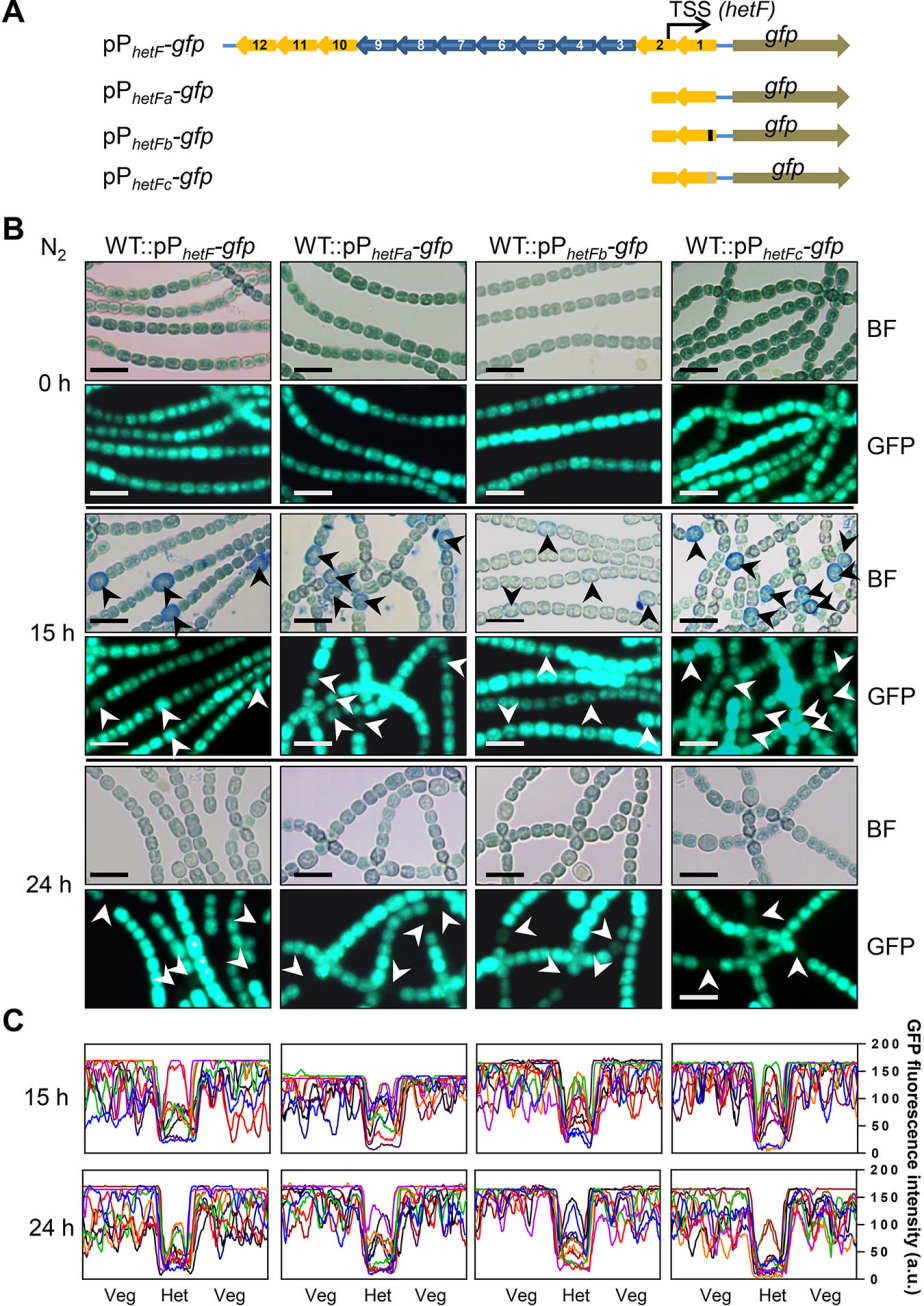
*hetF* is expressed in vegetative cells. (A) Sketch illustration of four transcription fusion constructs of *hetF*. The upstream *nsiR1* gene array is indicated by blue and yellow arrows, and genes with the same color are identical in sequence. The TSS (black arrows) of *hetF* is located in *nsiR1.2*. pP*_hetF_*-*gfp* includes the whole *nsiR1* array (*nsiR1.1* to *nsiR1.12*). pP*_hetFa_*-*gfp* includes only *nsiR1.1*. pP*_hetFb_*-*gfp* and pP*_hetFc_*-*gfp* are generated from pP*_hetFa_*-*gfp* with the −10 box being replaced (black box) and deleted (gray box), respectively. (B) Expression pattern of *hetF* during heterocyst formation. The fluorescence pattern of WT cells harboring pP*_hetF_*-*gfp*, pP*_hetFa_*-*gfp*, pP*_hetFb_*-*gfp*, or pP*_hetFc_*-*gfp* was captured after 0 h, 15 h, and 24 h of nitrogen (N_2_) step-down. BF, bright field. Cells were stained with Alcian blue for better visualization of proheterocysts at 15 h. Arrows indicate proheterocysts or mature heterocysts. Bars, 10 μm. (C) Quantification of GFP fluorescence in proheterocysts or heterocysts after 15 h and 24 h of nitrogen step-down. At each time point, the fluorescence signals along 10 representative short filaments, each of which consists of 1 heterocyst (Het) and 3 surrounding vegetative cells (Veg) on each side, were quantified with ImageJ. Every curve in the graphs represents the distribution of the fluorescence intensity along one filament. a.u., arbitrary units.

*hetF* expression was described as constitutive, but the experimental details were not provided (31). Transcriptome sequencing (RNA-seq) data indicate that the transcription start site (TSS) is located at bp −256 upstream of the *hetF* open reading frame (ORF) (34). *hetF* is preceded by an array of 12 small noncoding RNAs (*nsiR1.1* to *nsiR1.12*) that are identical or highly similar to each other, all transcribed in the opposite direction from *hetF*. All 12 copies of *nsiR1* have been shown to be activated early during heterocyst development following nitrogen step-down in an NtcA- and HetR-dependent manner (34–36). To investigate the cell type-specific expression of the *hetF* gene and the contribution of *nsiR1*, we made two *gfp* fusions with different promoters: a long version that covered the entire small RNA array (pP*_hetF_-gfp*) and a short version that included only the immediately surrounding region of the transcription start site (pP*_hetFa_-gfp*) (Fig. 1A). The replicative plasmid bearing each fusion was conjugated into *Anabaena*. As shown in Fig. 1B, in combined-nitrogen-replete medium with nitrate (BG11), strong green fluorescent protein (GFP) fluorescence was detected in both strains, without a noticeable difference. Proheterocysts could be identified by Alcian blue staining (37); we thus monitored the changes of the GFP fluorescence intensity and quantified the proheterocyst or heterocyst frequency during the process of heterocyst differentiation. At 15 h postinduction, the GFP signal in some proheterocysts was still high as in vegetative cells, while about 80% of proheterocysts that had been stained by Alcian blue had lower GFP signals (Fig. 1B and C). The proheterocyst frequencies in the two strains were 5.81% and 5.91%, respectively (see Fig. S1 in the supplemental material). At 24 h postinduction, the GFP signal in most heterocysts was lower than that in vegetative cells (Fig. 1B and C), and the heterocyst frequencies were 8.31% and 8.66%, respectively (Fig. S1). These frequencies were comparable to those of the wild type (WT) under similar conditions (Fig. S1). One copy of *nsiR1* (*nsiR1.1*) after the *hetF* TSS remained intact in pP*_hetFa_-gfp*; to inactivate this last copy, we either replaced the putative −10 box of *nsiR1.1* with a different sequence or removed it from pP*_hetFa_-gfp*, which generated pP*_hetFb_-gfp* and pP*_hetFc_-gfp*, respectively. We found that these changes led to little variation of the GFP fluorescence under both combined-nitrogen-replete or -depleted conditions (Fig. 1B and C), and so did the proheterocyst or heterocyst frequency after 15 h or 24 h of nitrogen step-down (Fig. S1). Our results indicate that *hetF* is transcribed in vegetative cells but downregulated in mature heterocysts (Fig. 1B and C). Such an expression pattern suggests a function of *hetF* in vegetative cells.

10.1128/mBio.01382-21.2 FIG S1 Proheterocyst or heterocyst frequencies in WT, WT::pP*_hetF_-gfp*, WT::pP*_hetFa_-gfp*, WT::pP*_hetFb_-gfp*, and WT::pP*_hetFc_-gfp* cells after 15 h and 24 h of nitrogen step-down. For each sample, 1,000 cells were analyzed. Download FIG S1, TIF file, 0.4 MB. Copyright © 2021 Xing et al. 2021 Xing et al. https://creativecommons.org/licenses/by/4.0/ This content is distributed under the terms of the Creative Commons Attribution 4.0 International license.

### The cell division defect of the *hetF* mutant is dependent on light intensity and the nitrogen regime.

**FIG 2:**
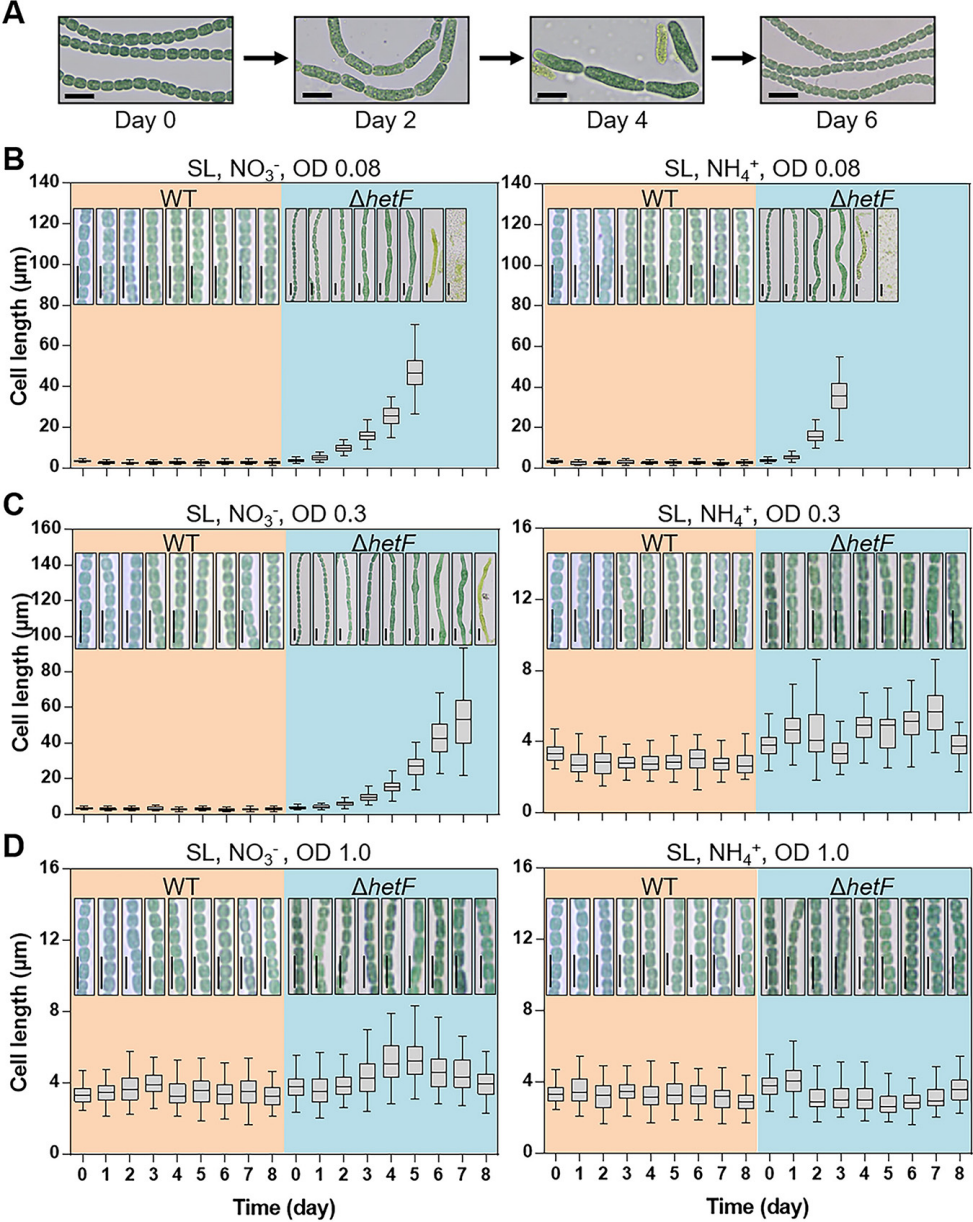
The cell filamentation phenotype of the Δ*hetF* mutant is dependent on light intensity and the nitrogen regime. (A) The cell division defect of the Δ*hetF* mutant changes in the culture. Images of Δ*hetF* cells were taken on different days during cultivation in standard light (SL). Bars, 10 μm. (B to D) Effects of cell optical density (OD) and nitrogen sources on the Δ*hetF* and WT cell lengths. Continuous growth of the cell cultures under the specified conditions (light intensity, nitrogen source, and OD values are indicated at the top of each panel) was monitored, and the OD was kept close to the original value by daily dilution with the respective fresh medium. One hundred cells were measured for each sample, and representative microscopic images are presented (all cultures were grown in SL) (for more details, see Materials and Methods). Note that cell lysis occurred with time under conditions of a low OD. Bars, 10 μm.

To investigate *hetF* functions, a markerless deletion mutant of *hetF* was created as described previously (38). As reported previously (30, 31), we confirmed that the Δ*hetF* mutant could not form heterocysts and consequently was unable to grow under diazotrophic conditions (Fig. S2). A dramatic cell division defect, with cell elongation and a slight increase in cell width, was observed under standard culture conditions for the mutant cells. However, this phenotype was not always reproducible, even for cells from the same culture. Thus, continuous monitoring of the mutant phenotype was performed, and the result showed that the cell division phenotype of the Δ*hetF* mutant changed throughout the growth course (Fig. 2A). With a fresh culture started at a low optical density (OD) of 0.08, cell filamentation appeared between day 2 and day 4, and the cell morphology then returned to normal, as for the WT, with increasing OD values (Fig. 2A).

10.1128/mBio.01382-21.3 FIG S2 The cell filamentation phenotype of the *hetF* mutant is dependent on the culture conditions. With a starting OD_750_ of 0.08, images of WT and Δ*hetF* cells were taken at days 2, 4, and 6 during cultivation under different light intensities (LL or SL) and nitrogen sources (NH_4_^+^ or NO_3_^−^). To test the differentiation of heterocysts, cells at day 2 using NH_4_^+^ as the nitrogen source were transferred into combined-nitrogen-free medium (N_2_) and incubated for a further 2 days, followed by observation under a microscope. Note that although cells in the culture from LL with ammonium have little phenotype in cell division, no heterocysts were induced. Arrows indicate heterocysts. Bars, 10 μm. Download FIG S2, TIF file, 1.4 MB. Copyright © 2021 Xing et al. 2021 Xing et al. https://creativecommons.org/licenses/by/4.0/ This content is distributed under the terms of the Creative Commons Attribution 4.0 International license.

**FIG 3:**
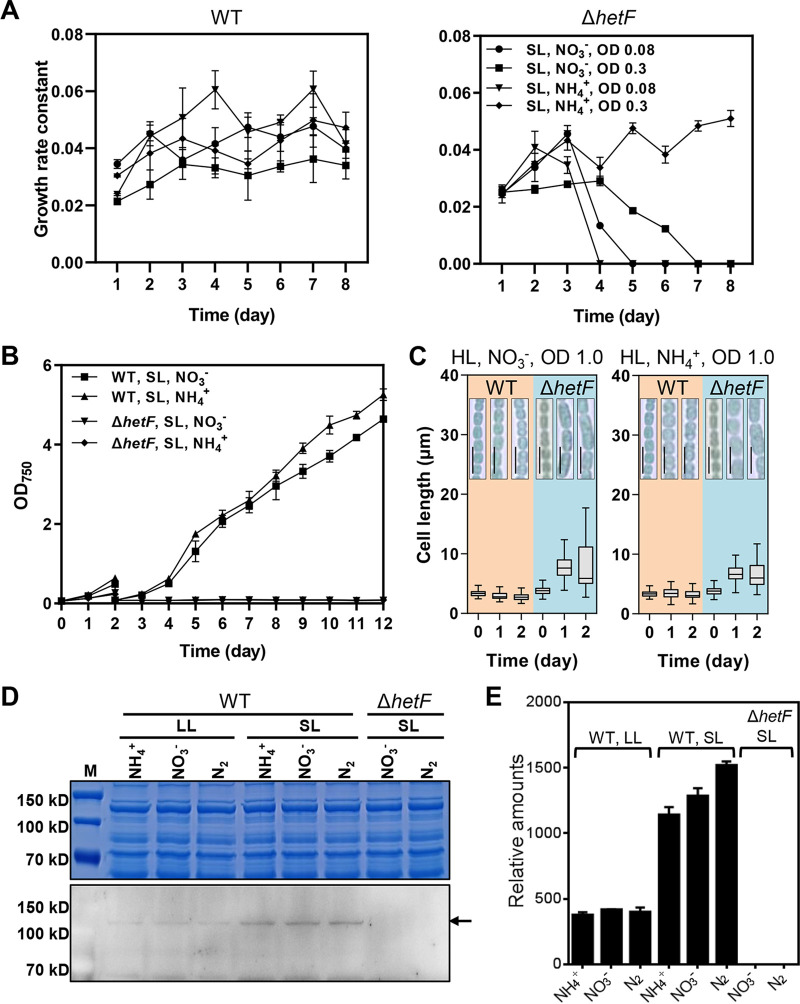
Effect of the nitrogen regime and cell density on the growth of WT and Δ*hetF* strains and the expression of HetF. (A) Growth rate constant analysis of the WT and Δ*hetF* strains. The cultures were maintained at a relatively stable optical density (OD) by daily dilution with fresh medium under the conditions described in the legends of Fig. 2B and C. (B) Effect of the nitrogen regime and cell density on the growth of WT and Δ*hetF* strains. Growth curves were measured by monitoring the OD with a spectrometer. With a starting OD_750_ of 0.08, the two strains were first incubated under SL in the presence of either nitrate or ammonium for 2 days. The cultures were then diluted back to an OD_750_ of 0.08 for better expression of the phenotype and cultured continuously. (C) Effect of nitrogen sources on the cell filamentation phenotype of WT and Δ*hetF* strains under high light (HL). Bars, 10 μm. One hundred cells were measured for each sample. HL, the nature of the nitrogen source, and the OD value (at 1.0) are indicated at the top of each panel. (D) Western blotting of HetF expression levels at day 2 under different conditions corresponding to Fig. S2 in the supplemental material using polyclonal antibody against the caspase HetF associated with tetratricopeptide repeats (CHAT) domain (aa 1 to 555) of HetF. M, molecular weight marker. kD, kilodalton. (E) Quantification of HetF protein levels based on the Western blots in panel D. Error bars represent means from three measurements with standard deviations.

We sought to determine the factors accounting for the variation of the cell division phenotype of the Δ*hetF* mutant. Since cells at a higher OD receive less light exposure due to the shading effect, we first investigated whether light intensity may be one such factor. We thus monitored the development of cell morphology with cultures maintained at low, medium, and high ODs with nitrate or ammonium as the nitrogen source and with daily dilution of the cultures back to the initial OD value. As shown in Fig. 2B, in the culture with nitrate, cells at a low OD of 0.08 quickly developed a cell division defect as early as day 2, and cells continuously elongated, reaching 27 μm to 75 μm in length, while WT cells had a cell length ranging from 1.6 μm to 4.2 μm (Fig. 2B, left). From day 6, extensive cell lysis occurred, and the culture collapsed (Fig. 2B, left), thus indicating that *hetF* was essential under such conditions. The essential function of *hetF* under such conditions was further confirmed by measuring the growth curves of the WT and the mutant (Fig. 3B). A similar process was found for cells maintained at a medium OD of 0.3, with slightly slower progression of the phenotype (Fig. 2C, left). However, cells grown and maintained at a high OD of 1.0 remained with a normal cell shape, as for the WT (Fig. 2D, left); however, when exposed to higher light intensity (HL), these cells again developed the cell division defect (Fig. 3C, left). These results indicate that increasing the intensity of illumination arrested cell division in the mutant, even at high OD values. As a control, WT cells cultured under similar light regimes showed little variation in cell length (Fig. 2B to D, left, and Fig. 3C, left).

In addition to light intensity, ammonium also appeared to affect the phenotype although less strongly. When ammonium was present as the nitrogen source, cells maintained at a low OD (0.08) showed a defect in cell division, but no significant cell filamentation was observed, even at a medium OD of 0.3 compared to cells growing with nitrate (Fig. 2B and C). Therefore, ammonium somehow suppressed the cell division phenotype with increasing OD values (Fig. 2B and C, right, and Fig. 3C, right). The effect of light and ammonium was unrelated to the cell growth rate because ammonium could support faster cell growth than nitrate (Fig. 3A) but nevertheless led to a much weaker phenotype in cell division (Fig. 2C). Furthermore, we monitored the phenotype with cultures under low light (LL) or standard light (SL) with different nitrogen sources. Our results showed that most of the cells grown under LL with ammonium as the nitrogen source have a normal cell shape (Fig. S2), while conditions under LL or SL with nitrate induced a cell division defect phenotype (Fig. S2).

Together, these results demonstrate that the phenotype of the Δ*hetF* mutant is dependent on the light intensity and nitrogen regime, providing a rationale for the inconsistency in the cell division phenotype of the mutant in the published data (30–33). This study also allowed us to optimize the culture conditions so that the function of *hetF* in cell division could be investigated consistently. For subsequent experiments, the *hetF* mutant was maintained under LL with ammonium where the cells could divide almost as normally as the WT (thus defined as permissive conditions) and then shifted to LL or SL with nitrate at a low OD (nonpermissive conditions) for phenotypic dissection.

Next, to better understand the conditional requirement of *hetF* in cell division, the level of HetF was examined by an immunoblot assay in the WT. The results showed that the HetF level was about 3-fold higher under SL than under LL conditions regardless of the nitrogen source used (Fig. 3D and E). Therefore, the level of HetF increased in cells under conditions where HetF is necessary for cell division.

### HetF localizes at midcell and cell-cell junctions.

**FIG 4:**
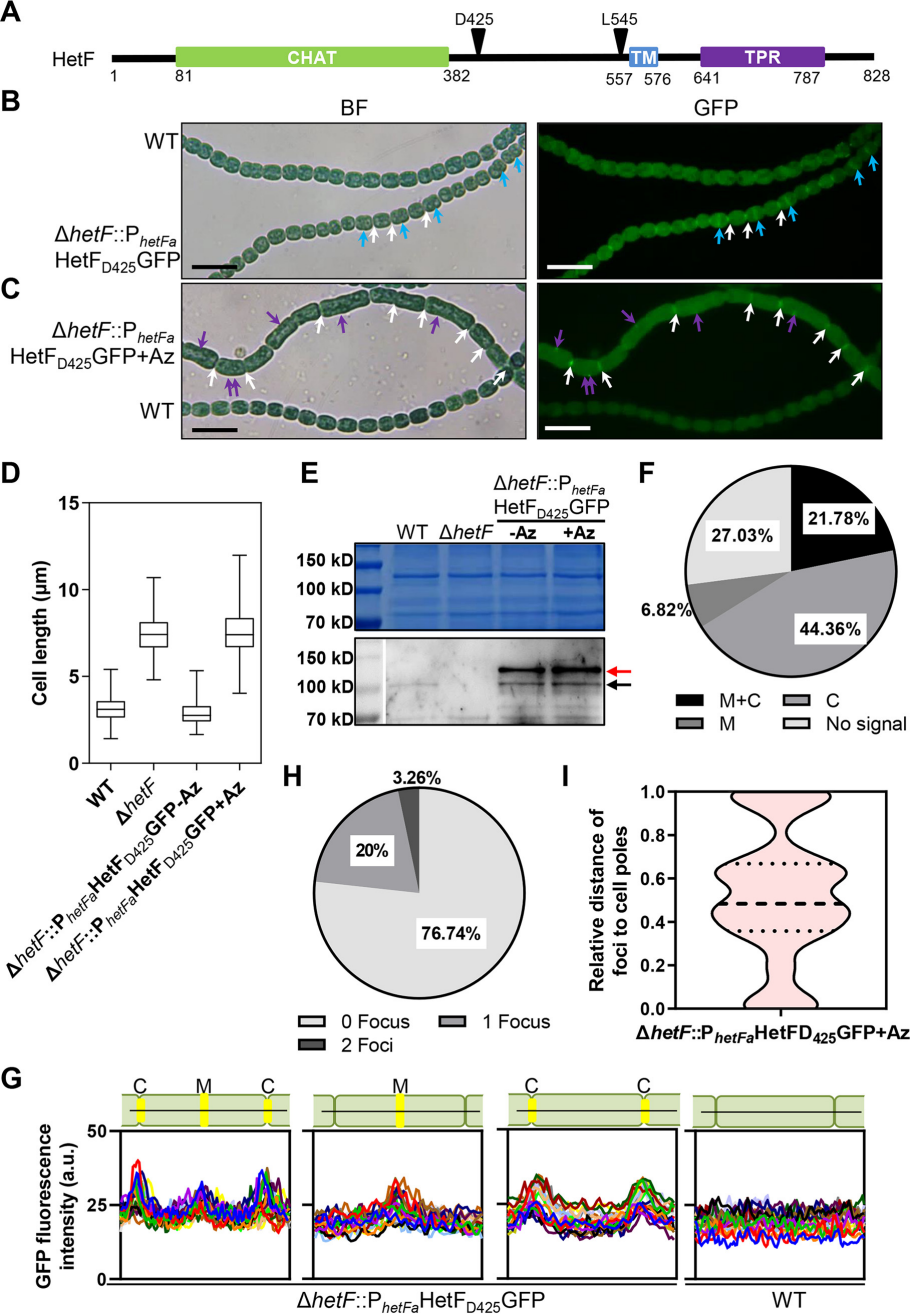
Subcellular localization of HetF in *Anabaena.* (A) Domain organization of HetF. CHAT, caspase HetF associated with tetratricopeptide repeats domain; TM, transmembrane domain; TPR, tetratricopeptide repeats domain. GFP insertion sites are indicated by arrows. (B) Subcellular localization of HetF_D425_GFP. Micrographs were taken with a mixed culture of Δ*hetF*::pP*_hetFa_*-HetF_D425_GFP and WT strains after 72 h of cultivation under LL in BG11 and with a starting OD_750_ of 0.08. Light blue and white arrows indicate the localization of HetF_D425_GFP at midcell and cell-cell junctions, respectively. (C) Micrographs of Δ*hetF*::pP*_hetF_*-HetF_D425_GFP cells after 72 h of cultivation in BG11 and treatment with 100 μM aztreonam (Az) under LL. Purple arrows indicate the HetF_D425_GFP foci at the sidewall of the cells. (D) Cell length analysis of the WT, Δ*hetF*, and Δ*hetF*::pP*_hetFa_*-HetF_D425_GFP strains with or without aztreonam after 72 h of cultivation under LL in BG11 and with a starting OD_750_ of 0.08. A total of 200 cells were measured for each sample. (E) Western blotting of HetF expression levels with the same samples as those in panel D using polyclonal antibody against the CHAT domain (aa 1 to 555) of HetF. The red arrow indicates HetF_D425_GFP expressed from a replicative plasmid, and the black arrow indicates HetF in the WT; a faint band was also detected in the complemented strain, possibly a degradation product of the fusion. (F) HetF_D425_GFP localization analysis in Δ*hetF*::pP*_hetFa_*-HetF_D425_GFP cells based on the same samples as the ones in panel B. A total of 378 cells were used for this analysis. M+C, proportion of cells that had a HetF_D425_GFP signal both at midcell and at cell-cell junctions; M, proportion of cells that had only a HetF_D425_GFP signal at midcell; C, proportion of cells that had only a HetF_D425_GFP signal at cell-cell junctions; No signal, proportion of cells that did not show a HetF_D425_GFP signal at midcell and cell-cell junctions. (G) Quantification of HetF_D425_GFP fluorescence in Δ*hetF*::pP*_hetFa_*-HetF_D425_GFP and WT cells corresponding to the same samples as the ones in panel B. The fluorescence signals along 20 representative cells for each type were quantified with ImageJ. Each line in the graphs represents the fluorescence intensity across the long axis of one cell. M, midcell; C, cell-cell junction. (H) Analysis of the number of HetF_D425_GFP foci in single cells of the Δ*hetF*::pP*_hetFa_*-HetF_D425_GFP strain from the same sample as the one in panel C. A total of 430 cells were used for analysis of the number of foci. Proportions of cells showing 0 foci, 1 focus, or 2 foci are shown. (I) Relative distance of foci to cell poles in the Δ*hetF*::pP*_hetFa_*-HetF_D425_GFP strain based on the same sample as the one in panel C. Cell length was considered 1 unit on the *y* axis (0 and 1 represent the two cell poles, and 0.5 represents the center of the cell). A total of 68 foci were used for analysis. BF, bright field; bars, 10 μm (B and C).

Many proteins involved in cell division display a midcell localization. We therefore examined the subcellular localization of HetF with the help of a GFP fusion. Initially, the GFP sequence was fused to either the N or C terminus of HetF with a flexible peptide linker. However, neither of the fusions gave rise to detectable fluorescence. HetF is a multidomain protein (Fig. 4A). We sought to minimize the interference of GFP folding in the fusion product. An analysis of the secondary structure using PSIPRED suggested the presence of two flexible regions in HetF, one from Q385 to C470 and another from C485 to W557 (Fig. S3). We therefore chose these two regions for making an in-frame insertion of *gfp*, at bp 1275 and bp 1635 (corresponding to D425 and L545, respectively), within the coding region of *hetF*. When the fusions were expressed in *Anabaena*, only the one with the GFP sequence inserted after D425 (HetF_D425_GFP, corresponding to the insertion of *gfp* at bp 1275) showed fluorescence (see below). This fusion was functional because a construct expressing HetF_D425_GFP under the control of the native promoter P*_hetFa_* complemented the Δ*hetF* mutant for both heterocyst development (Fig. S4A) and cell division (Fig. 4B and D) under conditions (LL with nitrate at a low OD) where the Δ*hetF* mutant still had the cell division arrest phenotype and a defect in heterocyst differentiation (Fig. S2). According to the results of Western blotting, HetF_D425_GFP was stably expressed, at a level even higher than that of the WT, possibly because it was expressed from a multicopy plasmid (Fig. 4E). A signal at the size of WT HetF was also found, likely a product of HetF_D425_GFP degradation since it also had a higher level than that of the WT, similarly to the full-length fusion protein. As expected, no signal corresponding to that found in the WT was detected in the Δ*hetF* mutant. This complemented strain allowed us to check the subcellular localization of HetF_D425_GFP by comparing it with a WT filament pictured in the same frame under a microscope. After quantification, 21.78% of the cells had a GFP signal both at midcell and at the cell-cell junctions, 44.36% of the cells had a GFP signal only at the cell-cell junctions, and 6.82% of the cells had a GFP signal only at midcell. No GFP signal could be observed at midcell or cell-cell junctions in 27.03% of the cells (Fig. 4F). The fluorescence level in the middle of the cells (M) was lower than that found at cell-cell junctions (C) (Fig. 4G, right). HetF was mainly localized in the middle of cells just before physical constriction became visible (Fig. 4B, light blue arrows) or at cell-cell junctions (white arrows). This localization pattern was similar to that observed after 7-hydroxycoumarin-amino-d-alanine (HADA) labeling that marked PG synthesis at the septa (39).

10.1128/mBio.01382-21.4 FIG S3 Secondary structure prediction of HetF using PSIPRED and GFP insertion sites, indicated by arrows, tested in this study. Download FIG S3, TIF file, 1.9 MB. Copyright © 2021 Xing et al. 2021 Xing et al. https://creativecommons.org/licenses/by/4.0/ This content is distributed under the terms of the Creative Commons Attribution 4.0 International license.

10.1128/mBio.01382-21.5 FIG S4 HetF_D425_GFP function and localization analysis in *Anabaena*. (A) Heterocyst differentiation of Δ*hetF*::pP*_hetFa_*-HetF_D425_GFP cells after cultivation for 2 days under LL in BG11_0_ (N_2_). Arrows indicate heterocysts. (B and C) HetF localizes on the cell membrane. In WT cells, HetF_D425_GFP expressed under the control of the CT promoter shows membrane localization (B), and removal of the transmembrane domain (GFPΔTM) causes delocalization of the protein into the cytoplasm (C). WT::pCT-HetF_D425_GFP and WT::pCT-GFPΔTM cells were cultivated with inducers (4 mM theophylline plus 2 μM CuSO_4_) for 2 days under SL in BG11 (NO_3_^−^). BF, bright field. Bars, 10 μm. Download FIG S4, TIF file, 2.0 MB. Copyright © 2021 Xing et al. 2021 Xing et al. https://creativecommons.org/licenses/by/4.0/ This content is distributed under the terms of the Creative Commons Attribution 4.0 International license.

HetF is predicted to contain one transmembrane (TM) segment (Fig. 4A). Consistent with this prediction, HetF_D425_GFP fluorescence was found mostly at the periphery of cells when overexpressed under the control of a synthetic inducible promoter (copper and theophylline [CT] promoter) (38, 40) (Fig. S4B). When the TM domain was deleted, the fluorescence was evenly distributed in the cytoplasm (Fig. S4C). We therefore conclude that HetF is a membrane protein localized at midcell and cell-cell junctions, consistent with the cell division phenotype of the Δ*hetF* mutant.

### HetF acts at the step of septal PG synthesis.

**FIG 5:**
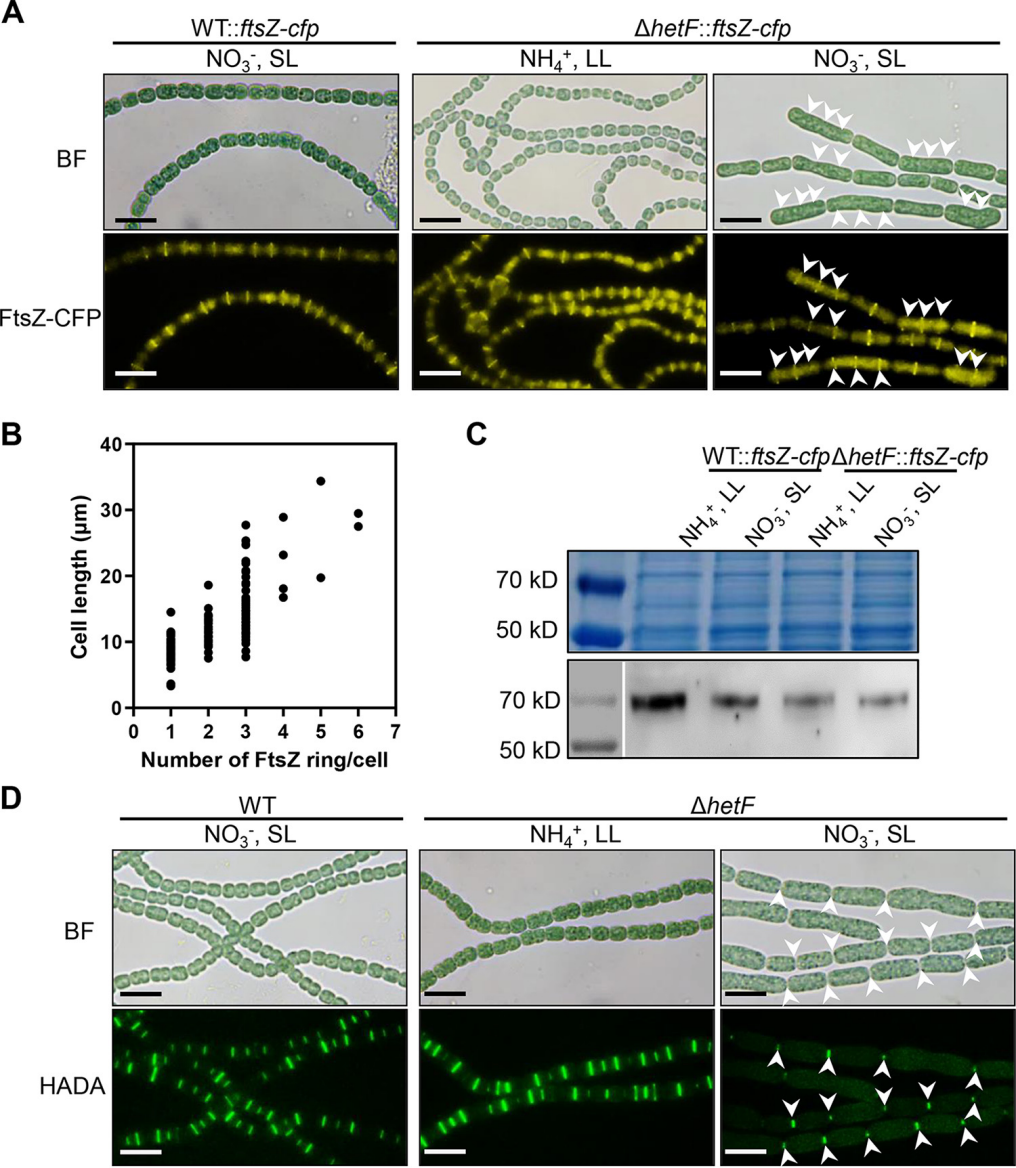
FtsZ ring formation and HADA labeling of PG synthesis. (A) FtsZ ring formation in the WT and Δ*hetF* strains. The cell division defect of the Δ*hetF*::*ftsZ-cfp* strain was induced by growing cells in BG11 (NO_3_^−^) under SL for 2 days. White arrows indicate the FtsZ-CFP rings in the elongated cells. (B) The number of FtsZ rings per cell is dependent on the cell length in the Δ*hetF* strain. One hundred forty cells were used for this analysis. (C) With similar amounts of total proteins loaded (top), Western blotting of FtsZ-CFP expression levels in the WT and the *hetF* mutant expressing *ftsZ-cfp* cultured with different nitrogen sources or light intensities using polyclonal antibody against GFP (bottom) was performed. (D) PG synthesis probed by HADA labeling. Cells were incubated with 200 μM HADA for 1 day under SL or 2 days under LL, using nitrate or ammonium as the nitrogen source, as indicated. White arrows indicate HADA fluorescence in the elongated cells. Bars, 10 μm (A and D).

During cell division, septal PG synthesis is driven by FtsZ through a treadmilling mechanism (7). First, we examined whether the deletion of *hetF* affected the localization of FtsZ. To do so, the native form of *ftsZ* was first replaced by an *ftsZ-cfp* fusion on the chromosome (WT::*ftsZ-cfp*), and *hetF* was then deleted on the chromosome by homologous recombination (Δ*hetF*::*ftsZ-cfp*). When the Δ*hetF*::*ftsZ-cfp* strain was cultured under LL in the presence of ammonium, a few cells on the filaments were slightly elongated compared to WT::*ftsZ-cfp* cells, but in most cells, FtsZ-cyan fluorescent protein (CFP) fluorescence was still found at midcell, similarly to WT::*ftsZ-cfp* cells (Fig. 5A). When the Δ*hetF*::*ftsZ-cfp* strain was cultured under SL with nitrate, extensive cell filamentation took place, a sign of cell division arrest; under such nonpermissive conditions, the Z-ring was still able to form, as one to several FtsZ-CFP ringlike structures were found in each elongated cell (Fig. 5A). After quantification, the number of FtsZ-CFP ringlike structures was found to be proportional to the cell length as cells elongated, although the amount of FtsZ-CFP in the WT::*ftsZ-cfp* strain detected by Western blotting was larger than that in the Δ*hetF*::*ftsZ-cfp* strain (Fig. 5B and C). Therefore, the deletion of *hetF* led to cell filamentation but did not affect FtsZ localization.

Next, we checked the effect of *hetF* deletion on the later steps of cell division, namely, septal PG remodeling. HADA is a fluorescence analogue of d-Ala that can be used to mark the site of septal PG synthesis in *Anabaena* filaments (39–41). In WT cells cultured either under LL with ammonium (data not shown) or under SL with nitrate, cell division occurred normally, and HADA fluorescence was observed at midcell or cell-cell junctions (Fig. 5D, left). A similar HADA fluorescence pattern was found when the Δ*hetF* mutant was grown under LL with ammonium. However, when the mutant was transferred to nonpermissive conditions, no HADA labeling could be found along the elongated cells, except those at the cell-cell junctions that represented the cell poles from the preceding cell division cycle (Fig. 5D, middle and right). This observation suggested that although FtsZ rings were still formed in the elongated cells, HADA incorporation and, hence, PG remodeling did not take place in the absence of HetF. Taken together, our results show that HetF is required for cell division at the step of septal PG synthesis.

### HetF interacts with FtsI, and this interaction is required for cell division.

**FIG 6:**
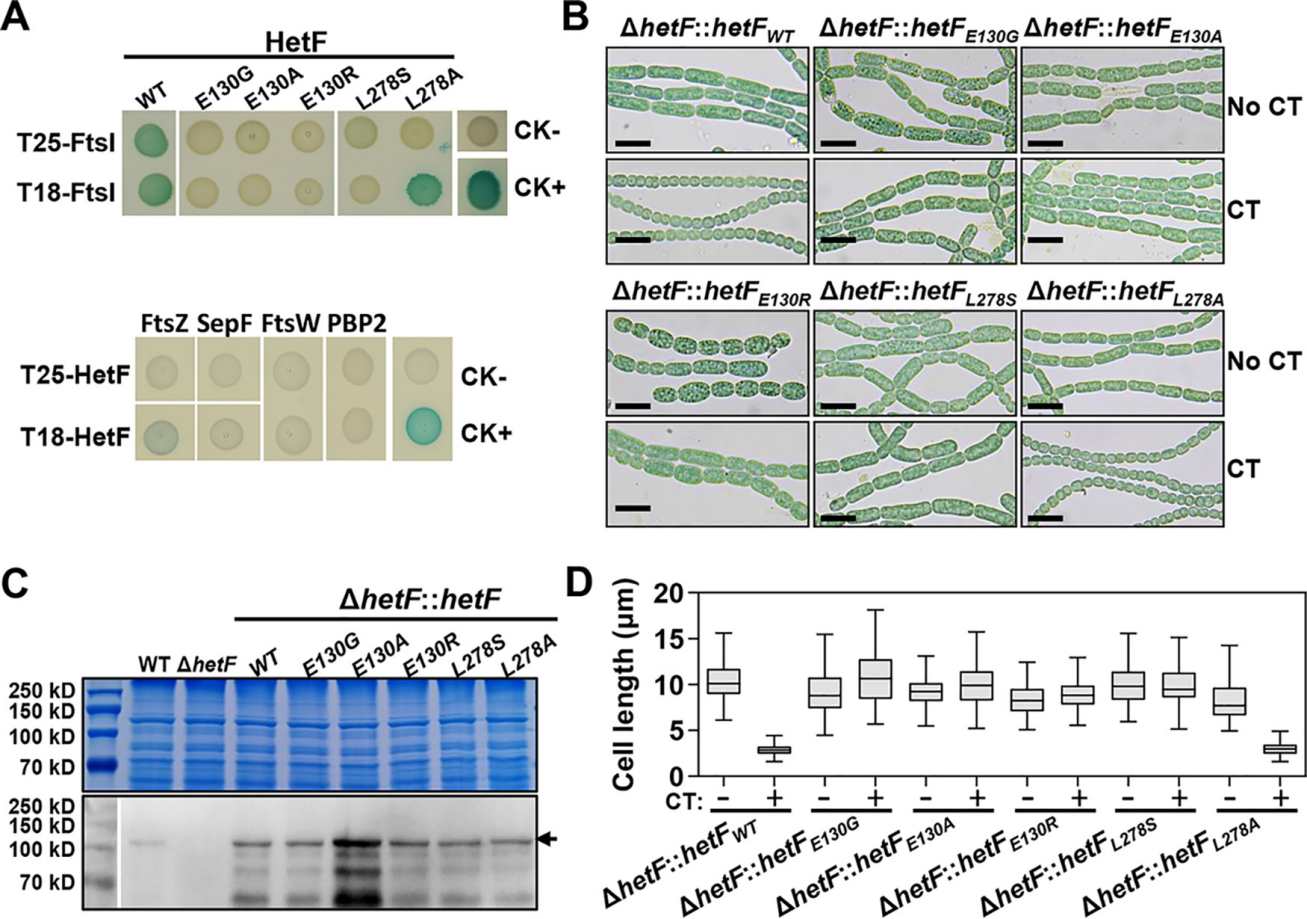
Interaction between HetF and FtsI is required for cell division. (A) Binary protein-protein interaction detected by BACTH assays for HetF (WT) or its variants with point mutations expressed using either the T25 or T18 vector. A positive interaction between the two targeted proteins is indicated by the blue color of the colony, while a negative interaction causes no change in cell color (for details, see Materials and Methods). CK− and CK+ indicate the negative and positive controls in the BACTH assays, respectively. (B) Mutant HetF proteins with point mutations unable to interact with FtsI fail to complement the cell division defect of the Δ*hetF* mutant (for details, see Materials and Methods), in contrast to the WT form of HetF. CT, copper and theophylline for induction of *hetF* expression. (C) Western blotting of HetF levels in the strains from panel B using polyclonal antibody against the CHAT domain (aa 1 to 555) of HetF. Total protein extracts (top) used for Western blotting (bottom) were used as the loading control. (D) Cell length analysis of the same strains as the ones in panel B. Two hundred cells were measured for each sample.

The localization of HetF at midcell and its requirement for septal PG synthesis suggest that HetF could be directly involved in cell division as a component of the divisome. As a major enzyme for PG synthesis in the divisome, FtsI/PBP3 is known to be a target of the antibiotic aztreonam, which inhibits septal cell wall synthesis (42). Previously, we found that impairment of FtsI by aztreonam led to cell filamentation and abolished PG synthesis at septal sites but did not affect FtsZ ring formation (39). This observed effect of aztreonam was therefore similar to that of *hetF* deletion and was further confirmed in this study (Fig. 5 and Fig. S5). We thus tested whether HetF could interact with FtsI. Using a bacterial two-hybrid (BACTH) system, we showed that HetF indeed interacted with FtsI when either of the partners was expressed on the two different vectors of the BACTH system (Fig. 6A, top). In contrast, no interaction was found between HetF and FtsZ, SepF, FtsW, or PBP2 (Fig. 6A, bottom).

10.1128/mBio.01382-21.6 FIG S5 FtsZ ring formation and HADA labeling of PG synthesis in the WT with the addition of the antibiotic aztreonam targeted at FtsI. (A) Micrographs of WT::*ftsZ-cfp* cells with aztreonam treatment. The WT::*ftsZ-cfp* strain was cultivated in BG11 (NO_3_^−^) with 100 μM aztreonam for 2 days under SL. White arrows indicate the FtsZ-CFP rings in two of the elongated cells. (B) Micrographs of WT cells labeled with HADA after aztreonam treatment. WT cells were cultivated in BG11 with 100 μM aztreonam for 1 day under SL, 200 μM HADA was then added, and cells were continuously cultivated for 1 day. White arrows indicate HADA fluorescence at cell-cell junctions. Bars, 10 μm (A and B). Download FIG S5, TIF file, 1.7 MB. Copyright © 2021 Xing et al. 2021 Xing et al. https://creativecommons.org/licenses/by/4.0/ This content is distributed under the terms of the Creative Commons Attribution 4.0 International license.

To further test the specificity and function of the FtsI-HetF interaction, we used the BACTH system to screen mutations that rendered HetF unable to interact with FtsI. After screening of a *hetF* mutant library generated by error-prone PCR, two point mutations of HetF, E130G and L278S, were identified, and HetF with either of the two mutations lost the ability to interact with FtsI (Fig. 6A, top). Further replacement of E130 with Ala or Arg (E130A and E130R) gave the same results, demonstrating that the E130 residue is crucial for the interaction of HetF with FtsI. On the other hand, when L278 of HetF was replaced by an Ala residue, this mutant form of HetF retained the ability to interact with FtsI but only when HetF_L278A_ was expressed from the T25 vector. To test the effect of such mutations *in vivo*, WT *hetF* or its different mutant versions were placed under the control of the inducible CT promoter and used in complementation assays of the Δ*hetF* mutant in *Anabaena*. Using WT *hetF* as a control, the Δ*hetF* mutant still showed a defect in cell division without induction by copper and theophylline (TP), but this defect was removed after the addition of inducers (Fig. 6B and D). In contrast, *hetF_E130R_*, *hetF_E130G_*, *hetF_E130A_*, or *hetF_L278S_* failed to complement the cell division defect of the Δ*hetF* mutant even after induction, in accordance with the inability of their corresponding proteins to interact with FtsI. Interestingly, *hetF_L278A_*, which encodes a HetF variant that is still able to interact with FtsI, retained the ability to complement Δ*hetF* (Fig. 6B and D). To rule out that the mutations making *hetF* nonfunctional are caused by protein stability, we checked the protein levels using polyclonal antibodies raised against HetF by Western blotting (Fig. 6C). Similar amounts of total proteins were loaded on the gel for different strains (Fig. 6C, top). HetF, with an apparent molecular weight of 91.8 kDa, was detected in the WT but not the Δ*hetF* mutant (Fig. 6C, bottom). In all strains, HetF mutant forms were detected at levels even higher than those of the WT, which is expected since these proteins were expressed from a replicative plasmid, pCT (38, 40). Therefore, the point mutations did not affect HetF stability. Together, these results demonstrate that the interaction of HetF with FtsI is essential for the function of HetF in cell division.

### Subcellular localization of HetF requires functional FtsI.

Since HetF is part of the divisome through interaction with FtsI, we examined whether a functional FtsI was required for the proper subcellular localization of HetF. For this purpose, the fluorescence of HetF_D425_GFP was examined in cells following the inhibition of FtsI by the addition of aztreonam (Fig. 4C). While the fluorescence of HetF_D425_GFP was still observed at cell-cell junctions formed after the preceding cell cycle, only one focus or two foci of fluorescence could be observed in 23.26% of elongated cells caused by inhibition with aztreonam (Fig. 4C and H). The distributions of HetF_D425_GFP fluorescence foci were not random along the cells as they were found mainly in between one-fifth and four-fifths of the cells or at positions close to the cell poles (Fig. 4I). These results indicate that once the function of FtsI was inhibited, HetF_D425_GFP could no longer be localized or stabilized at septa. Together with the interaction between HetF and FtsI, these results indicate that the recruitment of HetF to, or its stabilization at, the divisome is dependent on FtsI.

## DISCUSSION

In this study, we provide evidence that HetF participates directly in cell division in *Anabaena* as a member of the divisome depending on the environmental conditions. This conclusion is supported by several lines of evidence. First, *hetF* is expressed in vegetative cells under nitrogen-replete conditions (Fig. 1B). This expression pattern is different from those of genes critical for heterocyst development, such as *hetR*, *ntcA*, or *patS*, whose expression levels are low in vegetative cells but increased in developing cells and mature heterocysts (26–29, 43). Instead, the *hetF* expression profile is similar to those reported for genes involved in cell division, such as *ftsZ*, with downregulation in mature heterocysts (42, 44), consistent with the terminal nature of this cell type. Second, the Δ*hetF* mutant displays a dramatic cell filamentation phenotype, a clear sign of a cell division defect. This phenotype was the strongest when the mutant was cultured under conditions of increasing illumination with nitrate as a nitrogen source, and cells maintained under such conditions lysed following cell elongation, indicating that *hetF* was essential under such conditions. Low light intensity and, to a lesser extent, ammonium could suppress this phenotype. Third, HetF displays a midcell localization in cells ready to divide, and this localization persists until the end of cell division at the cell-cell junctions, as for some other cell division proteins and HADA labeling patterns in *Anabaena* (16, 39). In agreement with the subcellular localization, HetF interacts with FtsI as demonstrated by both the BACTH system and genetic evidence based on point mutations of HetF that disrupt the FtsI-HetF interaction. Furthermore, the *hetF* deletion mutant and a WT strain treated with aztreonam to inhibit FtsI give comparable results for FtsZ localization and HADA labeling. Finally, the proper subcellular localization of HetF requires a functional FtsI. Thus, HetF is recruited to or stabilized at the septum through its interaction with FtsI. When the interaction between FtsI and HetF is disrupted by adding the antibiotic aztreonam, HetF_D425_GFP foci are no longer associated with the divisome yet are still found mostly close to the center or cell-cell junctions (Fig. 4I), suggesting that they are still in relative proximity to the divisome complex.

Our data also clarified the inconsistency of cell division phenotypes of the *hetF* mutant in previous work (30–33). Although it is still unknown how light and nitrogen source affect the function of HetF, we observed that under conditions where HetF is required for cell division, the protein levels of HetF increase (Fig. 3D and E). On the other hand, the *hetF* mutant never differentiates into heterocysts, whether the cell division defect appeared or not (see Fig. S2 in the supplemental material). Thus, the conditional requirement of *hetF* is specific to its function in cell division, whereas its role in heterocyst development is essential.

Although major components involved in cell division are highly conserved in different bacteria, our study indicates that cyanobacteria have distinct features for their cell division machinery. The role of HetF in cell division may correspond to a mechanism for these organisms to adapt to the changing environment, such as light intensity, which is not only the energy source but also a shaping force for the whole physiology of photosynthetic organisms. Beyond the common features in bacterial cell division, the evolutionary history of particular bacteria leads to divergence and variation in cell division for better environmental adaptation.

## MATERIALS AND METHODS

The procedures for the construction of *Anabaena* mutant strains are described in detail in Text S1 in the supplemental material.

10.1128/mBio.01382-21.1 TEXT S1 Detailed procedures for the construction of *Anabaena* mutant strains. Download Text S1, DOCX file, 0.02 MB. Copyright © 2021 Xing et al. 2021 Xing et al. https://creativecommons.org/licenses/by/4.0/ This content is distributed under the terms of the Creative Commons Attribution 4.0 International license.

### Strains and growth conditions.

All strains used in this study are listed in Table S1. *Anabaena* strains were cultivated in BG11 (45) or BG11_0_ (BG11 without nitrate) in a shaker (30°C at 180 rpm) with standard light (SL) illumination of 30 μmol photons m^−2^ s^−1^. When necessary, cultures were incubated at low light (LL) illumination of 7 μmol photons m^−2^ s^−1^ or high light (HL) illumination of 70 μmol photons m^−2^ s^−1^. In order to maintain a stable cell division arrest phenotype, the Δ*hetF* culture was cultivated and stored in BG11_0_ with 2.5 mM NH_4_Cl under LL. The cell division arrest phenotype could be induced by cultivating cells in BG11 under SL with an initial OD at 750 nm (OD_750_) of 0.08 to 0.3 for 2 to 4 days. A total of 100 μg ml^−1^ neomycin or 5 μg ml^−1^ spectinomycin and 2.5 μg ml^−1^ streptomycin were added to the cultures as needed. All strains are listed in Table S1.

10.1128/mBio.01382-21.7 TABLE S1 Strains used in this study. Download Table S1, DOCX file, 0.02 MB. Copyright © 2021 Xing et al. 2021 Xing et al. https://creativecommons.org/licenses/by/4.0/ This content is distributed under the terms of the Creative Commons Attribution 4.0 International license.

### Assay for testing the effects of cell density, light intensity, and nitrogen sources on cell division arrest.

Δ*hetF* and WT cells were washed with BG11_0_ three times to remove NH_4_Cl and resuspended in BG11_0_ with 2.5 mM NH_4_Cl or BG11 to OD_750_ values of 0.08, 0.3, and 1, respectively. The cultures were then incubated under SL or HL. The OD value was measured every day, and the culture was then washed and diluted to the respective initial OD values again using the corresponding fresh medium. The growth rate constant was calculated according to the formula growth rate constant = 1/generation time = 3.322 × (log OD*_t_*_2_ − log OD*_t_*_1_)/(*t*_2_ − *t*_1_), where OD*_t_*_1_ is the OD_750_ of a culture 0 h after dilution and OD*_t_*_2_ is the OD_750_ of the same culture 24 h later (before dilution). Meanwhile, samples were collected every day for recording the phenotypes. The cell length was averaged from 100 or 200 cells.

### Testing the cell division arrest phenotype in the Δ*hetF* background.

Δ*hetF* background strains were washed with BG11 three times to remove NH_4_Cl before being resuspended in BG11 to an OD of 0.08 to 0.3. The cultures were incubated under SL for 2 days, and samples were then prepared for observing the cell division arrest phenotype. For complementation experiments, the cell division arrest phenotype of the Δ*hetF*::pCT-HetF strain was induced by cultivating cells in BG11 under SL for 2 days, and the cultures were then diluted to an OD of 0.08 to 0.3 in BG11 supplemented with 2 μmol CuSO_4_ and 4 mM theophylline and continuously incubated under SL for 1 to 2 days before recording the cell phenotypes. The same method was used for the strains expressing *hetF* point mutations, such as Δ*hetF*::pCT-HetFE130R.

### Multiclonal antibody preparation.

The plasmid pHTAlr3546CHATStrep was constructed by inserting a partial *hetF* fragment (bp 1 to 1665, amplified with primer pair Palr3546F1d/Palr3546R1665f) and a synthesized TwinStrep-tag fragment into pET28a. The expressed fusion protein contains the CHAT domain (amino acids [aa] 1 to 555) of HetF, a 6×His tag at its N terminus, and a TwinStrep-tag at its C terminus. To induce CHAT domain protein expression, E. coli BL21(DE3) cells containing pHTAlr3546CHATStrep were grown in LB medium with 0.5 μM isopropyl-β-d-thiogalactopyranoside (IPTG) at 37°C. After a 4-h induction, cells were collected and resuspended in lysis buffer (pH 7.4) (137 mM NaCl, 2.7 mM KCl, 8 mM Na_2_HPO_4_, 14.6 mM KH_2_PO_4_) and broken by a JN mini-low-temperature and ultrahigh-pressure cell breaker (JNBio Co. Ltd.). The lysate was centrifuged at 10,000 rpm for 40 min at 4°C. The precipitate (inclusion body) was washed twice with 2 M urea and then sent to Frabio Co. Ltd. for multiclonal antibody production.

### Preparation of *Anabaena* total protein and Western blot assay.

Cells were collected by filtration and then broken with a sample preparation system (FastPrep-24, 6.0 m/s; QuickPrep, 60 s) in LDS sample loading buffer (70 mM lithium dodecyl sulfate, 100 mM Tris-HCl [pH 8.5], 10% glycerol, 4 mM EDTA, 0.025% Coomassie brilliant blue G250). Cell extracts were boiled at 95°C for 10 min, followed by centrifugation at 135,000 rpm for 10 min. The supernatant was collected, loaded onto a 10% SDS-PAGE gel, and subsequently subjected to a Western blot assay with a multiclonal antibody against the CHAT domain in HetF.

### BACTH assay.

The bacterial adenylate cyclase two-hybrid (BATCH) system kit based on the reconstitution of adenylate cyclase was used for testing protein-protein interactions (46). *hetF*, *ftsI*, *ftsZ*, *sepF*, *ftsW*, and *pbp2* were amplified with primers listed in Table S2, and individual PCR products were assembled into linearized pUT18C and pKT25 vectors. All the resulting plasmids were verified by PCR and Sanger sequencing. The plasmids were cotransformed into strain BTH101, and the transformants were plated on solid LB medium containing 50 μg liter^−1^ ampicillin, 25 μg liter^−1^ kanamycin, 0.5 mM liter^−1^ IPTG, and 40 μg liter^−1^ 5-bromo-4-chloro-3-indolyl-β-d-galactopyranoside (X-gal).

10.1128/mBio.01382-21.8 TABLE S2 Primers used in this study. Download Table S2, DOCX file, 0.02 MB. Copyright © 2021 Xing et al. 2021 Xing et al. https://creativecommons.org/licenses/by/4.0/ This content is distributed under the terms of the Creative Commons Attribution 4.0 International license.

For screening of HetF point mutations that lost interaction with FtsI, the *hetF* ORF region was amplified using Green *Taq* mix (catalogue no. P131-AA; Vazyme Biotech Co. Ltd.) and primer pair Palr3546F1/Palr3546R2502 for 15 cycles. The PCR product was assembled into the pKT25 vector using a one-step cloning kit (ClonExpress II; Vazyme Biotech Co. Ltd.). The ligation product was transformed into BTH101 containing the T18-FtsI plasmid. White colonies that appeared on the plates were selected for colony PCR and checked for the presence of single missense mutations of HetF via Sanger sequencing.

### Microscopy.

An Sdptop EX30 microscope was used to take bright-field images, and an Sdptop EX40 epifluorescence microscope was used to take fluorescence images. A filter (exciter [EX] 379-401, dichroic beamsplitter [DM] 420LP, emitter [EM] 435-485) was used to image HADA fluorescence (exposure time of 200 ms). A filter (EX426-446, DM455LP, EM460-500) was used to image CFP fluorescence (exposure time of 1 s). A filter (EX470-490, DM495LP, EM500-520) was used to image GFP fluorescence (exposure time of 1 s). Fluorescence images were taken with an oil immersion lens objective (100×/1.28). All images were processed using ImageJ without deconvolution.

10.1128/mBio.01382-21.9 TABLE S3 Plasmids used in this study. Download Table S3, DOCX file, 0.03 MB. Copyright © 2021 Xing et al. 2021 Xing et al. https://creativecommons.org/licenses/by/4.0/ This content is distributed under the terms of the Creative Commons Attribution 4.0 International license.
