# Supplementary figures and images for: HetF Protein Is a New Divisome Component in a Filamentous and Developmental Cyanobacterium
Source: mBio. 2021 Jul 13;12(4):e01382-21. doi: 10.1128/mBio.01382-21 (PMC8406250; doi:10.1128/mBio.01382-21)

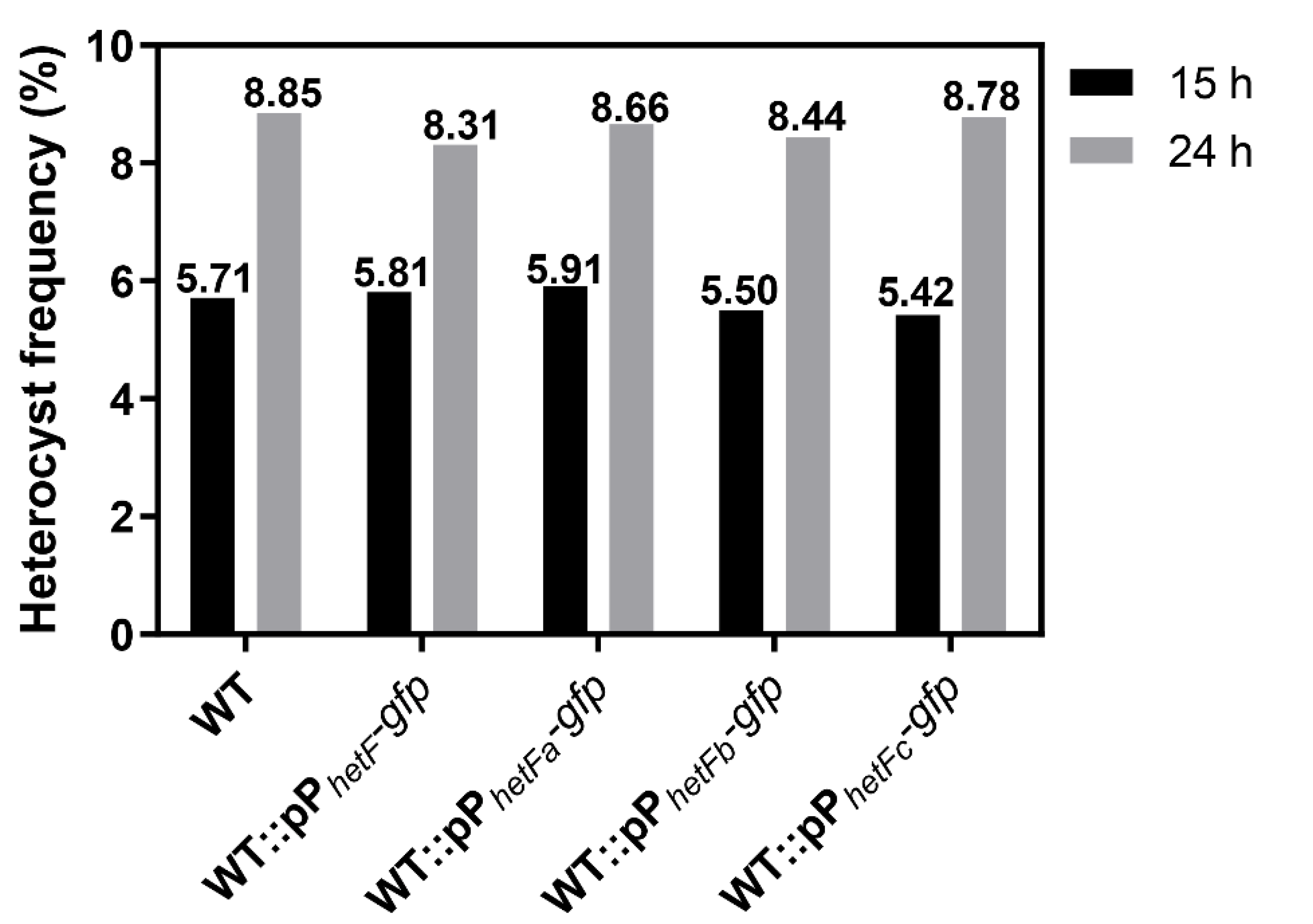

Supplement: FIG S1 [file mbio.01382-21-sf001.tif]

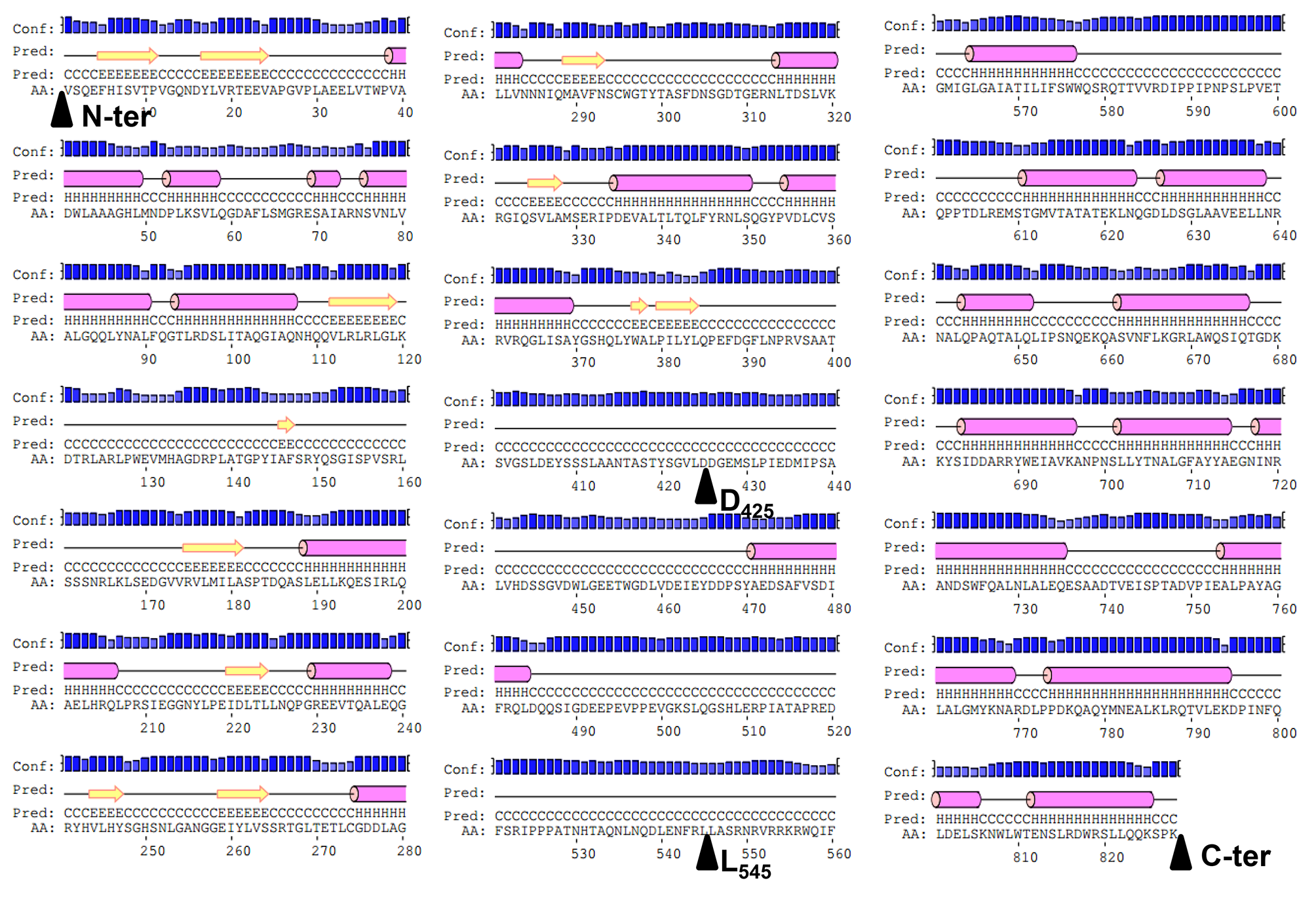

Supplement: FIG S3 [file mbio.01382-21-sf003.tif]

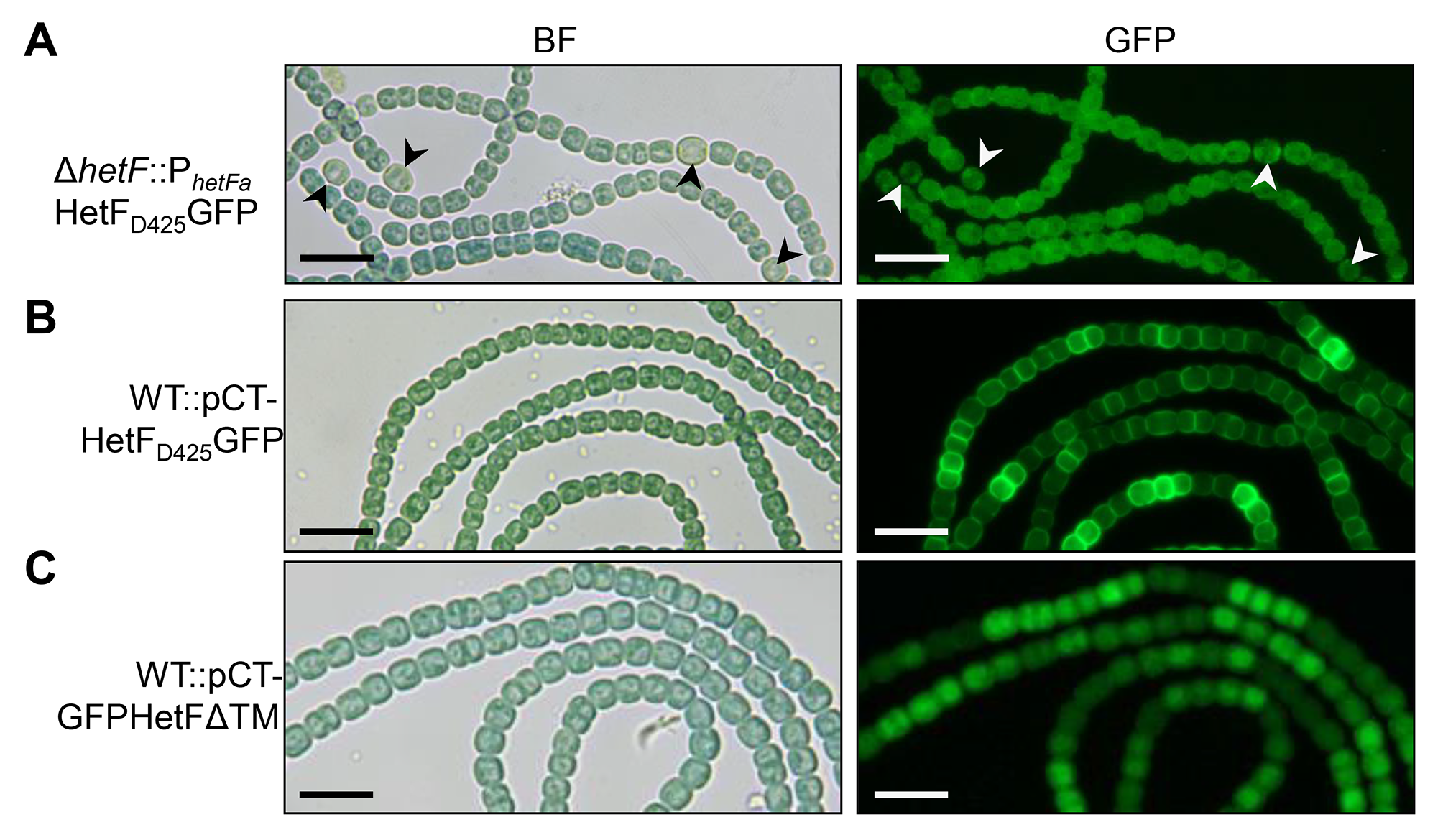

Supplement: FIG S4 [file mbio.01382-21-sf004.tif]

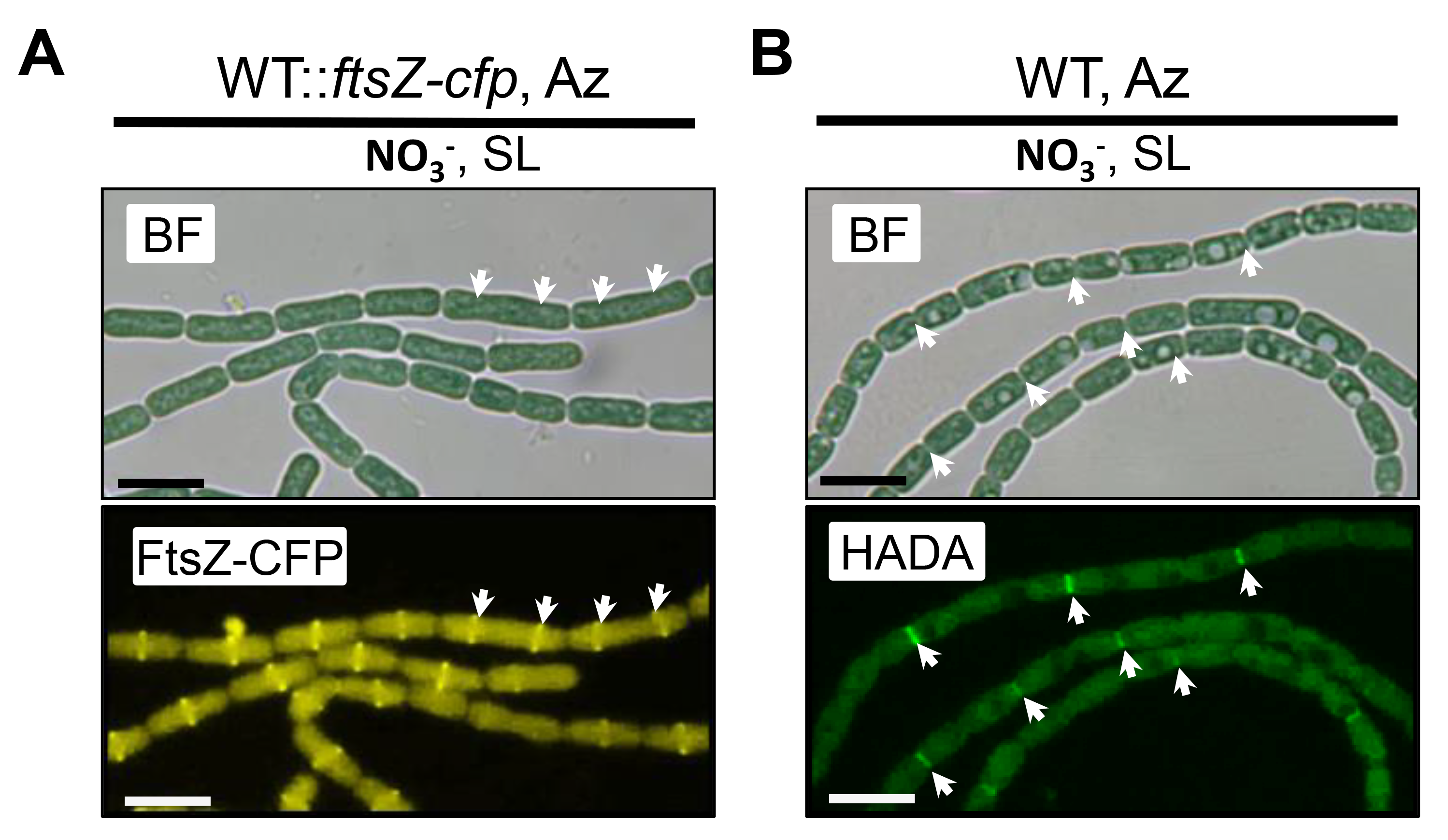

Supplement: FIG S5 [file mbio.01382-21-sf005.tif]
