# Supplementary material for: HetF Protein Is a New Divisome Component in a Filamentous and Developmental Cyanobacterium
Source: mBio. 2021 Jul 13;12(4):e01382-21. doi: 10.1128/mBio.01382-21 (PMC8406250; doi:10.1128/mBio.01382-21)
Supplement: TEXT S1 [file mbio.01382-21-t0001.docx]

SUPPLEMENTARY TEXT

**Construction of *Anabaena* mutant strains***. hetF* was constructed in a similar way as previously described using pCfp1 plasmid (1). The repair template was generated by fusing the upstream and downstream region of the target sequence using primers listed in Table S2. The spacer sequence was designed according to the described rules (1) and prepared by annealing two complementary primers. To generate the plasmid that knocks out a specific gene, the respective repair template and the spacer sequence were sequentially cloned into pCpf1 at the sites of *Bgl*II-*BamH*I and *Aar*I−*Aar*I.

To construct *gfp* transcriptional reporter strains for *hetF*, the promoter regions (−2276 to 30 for P***_hetF_***-*gfp* and −300 to 30 for P***_hetFa_***-gfp) of *hetF* were amplified with specific primers and subsequently cloned into *BamH*I-*Xho*I digested plasmid pRL25N-L*gfp* (2), resulting in plasmids pP***_hetF_***-*gfp* and pP***_hetFa_***-*gfp*. The plasmid pP***_hetFb_***-*gfp* is a derivative of pP***_hetFa_***-*gfp* bearing a mutation in the -10 box of *nsiR1*. This mutation is created via site-directed mutagenesis on pP***_hetFa_***-*gfp* using a pair of primers (Palr3546F166m and Palr3546R173m) that have the desired mutation in the overlapping sequence. pP***_hetFc_***-gfp was also derived from pP***_hetFa_***-*gfp,* through deletion of the -10 box of *nsiR1* using the same strategy.

To overexpress HetF using a CT promoter, the ORF region of *hetF* (1 to 2484 with respect to the start codon) amplified using the primers Palr3546F1f and Palr3546R2484e, was cloned into pCT at the sites of *Xho*I-*Sma*I (1, 3), resulting in the overexpression plasmid pCT-HetF. Plasmids bearing HetF variants with point mutations, pCT-HetFE130R, pCT-HetFE130G, pCT-HetFE130A, pCT-HetFL278S and pCT-HetFL278A, were constructed by site-directed mutagenesis using pCT-HetF as the template and respective primer pairs containing desired mutation (Palr3546E130R-seqF / Palr3546E130R-seqR, Palr3546E130G-seqF / Palr3546E130G-seqR, Palr3546E130A-seqF / Palr3546E130A-seqR, Palr3546L278S-seqF / Palr3546L278S-seqR, and Palr3546L278A-seqF / Palr3546L278A-seqR, respectively).

To check HetF localization in *Anabaena*, two translational fusions under different promoters were constructed using a similar strategy as described above. For the plasmid with native promoter, the repair template was generated by fusing 3 fragments (−300 to 1275 bp relative to *hetF* coding region, *gfp*, and 1276 to 2484 bp of *hetF*) by overlapping PCR. The repair template was sequentially cloned into pCT at the sites of *BamH*I-*Sma*I (1, 3), resulting in the translational fusion plasmid pP*_hetF_*-HetF_D425_GFP. The plasmid with CT promoter (for over-expression) was constructed in the same way, but using a repair template without the native promoter sequence (1 to 1275 bp of *hetF*, *gfp*, and 1276 to 2484 bp of *hetF*). The repair template was cloned into pCT at the sites of *Xho*I-*Sma*I (1, 3), resulting in the translational fusion plasmid pCT- HetF_D425_GFP.

Plasmid pCT-GFPHetFΔTM was used for checking the localization of a HetF variant without the TM domain*.* The repair template was generated by fusing 3 fragments (*gfp*, 1 to 1665 bp of *hetF*, 1732 to 2484 bp of *hetF*) using overlapping PCR. The repair template was cloned into pCT at the site of *Xho*I-*Sma*I (1, 3), resulting in the translational fusion plasmid pCT-GFPHetFΔTM.

Plasmid pRLAlr3858-CFP for checking the FtsZ localization in *Anabaena* was constructed with the suicide vector pRL277 (2). The repair template was generated by fusing 4 fragments (161 to 1284 bp of *ftsZ*, *cfp*, Km-resistant cassette, 1353 to 2375 bp of *ftsZ* coding region) using overlapping PCR. The repair template was then cloned into *Bgl*II-*Xho*I cutted pRL277 (2), resulting in the translational fusion plasmid pRLAlr3858-CFP.

To make the *Anabaena* mutant strains, the constructed plasmid (see Table S3) was transferred into *Anabaena* by conjugation (4, 5). The exconjugants were selected on BG11 plates containing appropriate antibiotics and subsequently verified by PCR and Sanger sequencing.

**REFERENCES**

1. Niu T-C, Lin GM, Xie LR, Wang ZQ, Xing WY, Zhang JY, Zhang CC. 2018. Expanding the potential of CRISPR-Cpf1 based genome editing technology in the cyanobacterium *Anabaena* PCC 7120. ACS Synth Biol 8:170-180. https://doi.org/10.1021/acssynbio.8b00437.

2. Zhang SR, Lin GM, Chen WL, Wang L, Zhang CC. 2013. ppGpp metabolism is involved in heterocyst development in the cyanobacterium *Anabaena* sp. strain PCC 7120. J Bacteriol 195:4536–4544. https://doi.org/10.1128/JB.00724-13.

3. Xing WY, Xie LR, Zeng X, Yang Y, Zhang CC. 2020. Functional dissection of genes encoding DNA polymerases based on conditional mutants in the heterocyst-forming cyanobacterium *Anabaena* PCC 7120. Front Microbiol 11:1108. https://doi.org/10.3389/fmicb.2020.01108.

4. Cai YP, Wolk CP. 1990. Use of a conditionally lethal gene in *Anabaena* sp. strain PCC 7120 to select for double recombinants and to entrap insertion sequences. J Bacteriol 172:3138–3145. https://doi.org/10.1128/jb.172.6.3138-3145.1990.

5. Elhai J, Vepritskiy A, Muro-Pastor AM, Flores E, Wolk CP. 1997. Reduction of conjugal transfer efficiency by three restriction activities of *Anabaena* sp. strain PCC 7120. J Bacteriol 179:1998–2005. https://doi.org/0.1128/jb.179.6.1998-2005.1997.
