# Supplementary material for: HetF Protein Is a New Divisome Component in a Filamentous and Developmental Cyanobacterium
Source: mBio. 2021 Jul 13;12(4):e01382-21. doi: 10.1128/mBio.01382-21 (PMC8406250; doi:10.1128/mBio.01382-21)
Supplement: TABLE S1 [file mbio.01382-21-st001.docx]

SUPPLEMENTARY TABLE S1

TABLE S1 Strains used in this study.

| Strains | Description | Source |
| --- | --- | --- |
| *Anabaena* PCC 7120 | Wild type | Pasteur Culture Collection |
| WT::pP*_hetF_*-gfp | Wild type bearing pP*_hetF_*-gfp; Nm^r^ | This study |
| WT::pP*_hetFa_*-gfp | Wild type bearing pP*_hetFa_*-gfp; Nm^r^ | This study |
| WT::pP*_hetFb_*-gfp | Wild type bearing pP*_hetFb_*-gfp; Nm^r^ | This study |
| WT::pP*_hetFc_*-gfp | Wild type bearing pP*_hetFc_*-gfp; Nm^r^ | This study |
| Δ*hetF* | *hetF* markless deletion mutant | This study |
| Δ*hetF*::pCT-HetF | Δ*hetF* bearing pCT-HetF; Nm^r^ | This study |
| Δ*hetF*::pCT-HetFE130R | Δ*hetF* bearing pCT-HetFE130R; Nm^r^ | This study |
| Δ*hetF*::pCT-HetFE130G | Δ*hetF* bearing pCT-HetFE130G; Nm^r^ | This study |
| Δ*hetF*::pCT-HetFE130A | Δ*hetF* bearing pCT-HetFE130A; Nm^r^ | This study |
| Δ*hetF*::pCT-HetFL278S | Δ*hetF* bearing pCT-HetFL278S; Nm^r^ | This study |
| Δ*hetF*::pCT-HetFL278A | Δ*hetF* bearing pCT-HetFL278A; Nm^r^ | This study |
| WT::*ftsZ-cfp* | *ftsZ* translational fusion with *cfp* on chromosome; Nm^r^ | This study |
| Δ*hetF*::f*tsZ-cfp* | *hetF* markless deletion mutant and *ftsZ* translational fusion with *cfp* on chromosome; Nm^r^ | This study |
| Δ*hetF*::pP*_hetF_*-HetF_D425_GFP | Δ*hetF* bearing pP*_hetF_*-HetF_D425_GFP; Nm^r^ | This study |
| Δ*hetF*::pCT- HetF_D425_GFP | Δ*hetF* bearing pCT-HetF_D425_GFP; Nm^r^ | This study |
| Δ*hetF*::pCT-GFPHetFΔTM | Δ*hetF* bearing pCT-GFPHetFΔTM; Nm^r^ | This study |
