# Supplementary material for: HetF Protein Is a New Divisome Component in a Filamentous and Developmental Cyanobacterium
Source: mBio. 2021 Jul 13;12(4):e01382-21. doi: 10.1128/mBio.01382-21 (PMC8406250; doi:10.1128/mBio.01382-21)
Supplement: TABLE S2 [file mbio.01382-21-st002.docx]

SUPPLEMENTARY TABLE S2

**TABLE S2** Primers used in this study.

| Primer | Sequence (5’-3’) |
| --- | --- |
| Palr3546F300m | GCAGAAATTCGATATCTAGATCGGGAATTGGTAAATTTTCCCTG |
| Palr3546R194 | AGCGCTACCGACGCTAGTGCT GAAAGAAACGCATCTCCCTGT |
| Palr3546F2501d | AGCACTAGCGTCGGTAGCGCT GAGAGATTGGGGAGTGGAGA |
| Palr3546R3382 | CGCAACGTTGTTGCCATTGCTGAGTAATTGCTGAGTGTAGTCA |
| cr_alr3546R848F | AGATACCAACAAACCCGCTAAATCGT |
| cr_alr3546R848R | AGACACGATTTAGCGGGTTTGTTGGT |
| Palr3546F2276m | ACATGGATCCTGTAGCTCTGTGTCTCTTG |
| Palr3546R30 | CACATCTCGAGGGTTACAGAAATGTGAAATTCC |
| Palr3546F300ma | ACATGGATCCGGGAATTGGTAAATTTTCCCTG |
| Palr3546F166m | CGTTAAACACAAGAGGGCAGATGCTAACCAATC |
| Palr3546R173m | CTGCCCTCTTGTGTTTAACGGTAGATGCACCTTGA |
| Palr3546F163m | AAACCCCCAGTGGCTCAGATGCTAACCAATCCGGATAA |
| Palr3546R221m | GCATCTGAGCCACTGGGGGTTTTTTCTATAACTAGCA |
| Palr3546F300mc | CGGCGGGGTTTTTTTTTGGA GGGAATTGGTAAATTTTCCCTG |
| Palr3546R2484e | ACCACCAGAACCCCC CTTGGGGCTTTTTTGTTGCAGA |
| Palr3546F1276c | GTGGTAGCACTAGCGTCGGTGATGGGGAAATGTCTTTACCGAT |
| Palr3546R1275c | GAGGCCTTGGATCCAGTCATATCCAGCACACCAGAGTAAGTAG |
| PYFP2-seF | ATGACTGGATCCAAGGCCTCT |
| PYFP2-seR | ACCGACGCTAGTGCTACC |
| Palr3546F1f | GAGGTAACAACAAGATGGTGTCCCAGGAATTTCACATT |
| Palr3546F1p | GTGGTAGCACTAGCGTCGGTGTGTCCCAGGAATTTCACATTTC |
| Palr3546R1665c | TGTCTGGCGACTTTGCTTGCGACGGACACGATT |
| Palr3546F1732b | CGTGTCCGTCGCAAGCAAAGTCGCCAGACAACAGTAG |
| Palr3546E130R-seqF | AGCACGTCTGCCGTGGCGAGTGATGCACGCAGGCGAT |
| Palr3546E130R-seqR | CGCCTGCGTGCATCACTCGCCACGGCAGACGTGCTAAC |
| Palr3546E130G-seqF | AGCACGTCTGCCGTGGGG AGTGATGCACGCAGGCGAT |
| Palr3546E130G-seqR | CGCCTGCGTGCATCACTC CCCACGGCAGACGTGCTAAC |
| Palr3546E130A-seqF | AGCACGTCTGCCGTGGGCAGTGATGCACGCAGGCGAT |
| Palr3546E130A-seqR | CGCCTGCGTGCATCACTGCCCACGGCAGACGTGCTAAC |
| Palr3546L278S-seqF | CCTCTGTGGCGACGATTCAGCGGGTTTGTTGGTTAAC |
| Palr3546L278S-seqR | TAACCAACAAACCCGCTGAATCGTCGCCACAGAGGGTT |
| Palr3546L278A-seqF | CCTCTGTGGCGACGATGCAGCGGGTTTGTTGGTTAAC |
| Palr3546L278A-seqR | TAACCAACAAACCCGCTGCATCGTCGCCACAGAGGGTT |
| Palr3546R1665f | GCTACCACCACCAGAACC CTTGCGACGGACACGATT |
| Palr3546F1d | TGCCGCGCGGCAGCCAT GTGTCCCAGGAATTTCACATT |
| Palr3858F161 | GCAGAAATTCGATATCTAGATCGTATTGGCGAGATTGTTCCTGG |
| Palr3858R1284 | TCCACCAGAGGCCTTGGATCCATTTTTGGGTGGTCGCCGTC |
| Palr3858F1353 | ACCGGATCATCAGTACTCCCTGCTAATTTTCAAGTTCAGAGGT |
| Palr3858R2375 | CGCAACGTTGTTGCCATTGCAGGTAGAACTTGTACCAGTGCA |
| PgfpspF | GGATCCAAGGCCTCTGGTGGATCTG |
| PgfpspRa | GGGAGTACTGATGATCCGGTGATT |
| Palr0718F1c | ATCACCTCTAGTGGTGAAATGCAAAAGTCACCAAGTAGAT |
| Palr0718R1830 | GATGTCGATCTAGATCTCTTAAGGTTTTCCTTCAATCGGCT |
| Palr3546F1 | ATCACCTCTAGTGGTGAAGTGTCCCAGGAATTTCACATT |
| Palr3546R2502 | GATGTCGATCTAGATCTCTCATCTTCCCGTACTCTACTT |
| Palr3858F1 | ATCACCTCTAGTGGTGAAATGACACTTGATAATAACCAAGAG |
| Palr3858R1307 | GATGTCGATCTAGATCTCAATCTCAAATCTAAAACCGCTTA |
| Palr0487F1 | ATCACCTCTAGTGGTGAAATGAACAATATATTTTCTAAACTGCGA |
| Palr0487R597 | GATGTCGATCTAGATCTCTTATTGTGCCATCCGGTT |
| Pall0154F1 | ATCACCTCTAGTGGTGAAGTGAAGCTACGCAGCCTAATTCC |
| Pall0154R1191 | GATGTCGATCTAGATCTCTTAAAACATCCGCCGACGTTG |
| Palr5101F1 | ATCACCTCTAGTGGTGAAATGCAATTCATATCTCTATCGCTTAC |
| Palr5101R2315 | GATGTCGATCTAGATCTCGGCTTTAGAAATTTCTTTCACAG |
