# Supplementary material for: HetF Protein Is a New Divisome Component in a Filamentous and Developmental Cyanobacterium
Source: mBio. 2021 Jul 13;12(4):e01382-21. doi: 10.1128/mBio.01382-21 (PMC8406250; doi:10.1128/mBio.01382-21)
Supplement: TABLE S3 [file mbio.01382-21-st003.docx]

SUPPLEMENTARY TABLE S3

TABLE S3 Plasmids used in this study.

| Plasmid | Description*^a^* | Source |
| --- | --- | --- |
| pCT | Km^r^Nm^r^; | 1, 3 |
| pCpf1-sp | Sm^r^ Sp^r^; CRISPR-Cpf1-Based Genome Editing vector | 1 |
| pCpf1 | Km^r^ Nm^r^; CRISPR-Cpf1-Based Genome Editing vector | 1 |
| pSfgfp-Sp | Sm^r^ Sp^r^; carrying supper foldding *gfp* coding sequence | 2 |
| pRL277 | Km^r^Nm^r^; | 2 |
| pBAD-mTurquoise2 | Km^r^Nm^r^; carrying cfp coding sequence | Addgene number: http://www.addgene.org/54844/ |
| pRL25N-Lgfp | Km^r^ Nm^r^; carrying *gfp* coding sequence | 2 |
| pCpf1-alr3546R848-sp | Sm^r^ Sp^r^; CRISPR-Cpf1 editing plasmid for *hetF* markless deletion | This study |
| pP*_hetF_*-gfp | Km^r^ Nm^r^; pRL25N-Lgfp carrying *hetF* promoter ( -2276 to 30 with respect to the start codon of hetF) and *gfp* coding gene | This study |
| pP*_hetFa_*-gfp | Km^r^ Nm^r^; pRL25N-Lgfp carrying *hetF* promoter ( -300 to 30 with respect to the start codon of hetF) and *gfp* coding gene | This study |
| pP*_hetFb_*-gfp | Km^r^ Nm^r^; the - 10 box of *nsiR1* that existed in *hetF* promoter based on pP*_hetFa_*-gfp had been mutant | This study |
| pP*_hetFc_*-gfp | Km^r^ Nm^r^; the - 10 box of *nsiR1* that existed in *hetF* promoter based on pP*_hetFa_*-gfp had been deleted | This study |
| pCT-HetF | Km^r^ Nm^r^; pCT carrying *hetF* ORF and CT promoter, used for over-expression | This study |
| pCT-HetFE130R | Km^r^ Nm^r^; pCT carrying *hetF* ORF with E130R point mutation and CT promoter, used for over-expression | This study |
| pCT-HetFE130G | Km^r^ Nm^r^; pCT carrying *hetF* ORF with E130G point mutation and CT promoter, used for over-expression | This study |
| pCT-HetFE130A | Km^r^ Nm^r^; pCT carrying *hetF* ORF with E130A point mutation and CT promoter, used for over-expression | This study |
| pCT-HetFL278S | Km^r^ Nm^r^; pCT carrying *hetF* ORF with L278S point mutation and CT promoter, used for over-expression | This study |
| pCT-HetFL278A | Km^r^ Nm^r^; pCT carrying *hetF* ORF with L278A point mutation and CT promoter, used for over-expression | This study |
| pP*_hetF_*-HetF_D425_GFP | Km^r^ Nm^r^; pCT carrying *hetF* ORF with gfp fusion and native promoter, used for hetF localization; gfp was inserted after 1245 with respect to the start codon of *hetF* | This study |
| pCT- HetF_D425_GFP | Km^r^ Nm^r^; pCT carrying *hetF* ORF with gfp fusion and CT promoter, used for *hetF* localization and over-expression; gfp was inserted after 1245 with respect to the start codon of *hetF* | This study |
| pCT-GFPHetFΔTM | Km^r^ Nm^r^; pCT carrying *hetF* ORF without TM domain (1666-1731) with gfp fusion and CT promoter, used for ΔTM localization and over-expression; gfp was inserted at the N-terminal of ΔTM | This study |
|  |  |  |
| pHTHetFCHATStrep | Km^r^; pHTwinStrep carrying *hetF* ORF (1-1665), used for protein expression and purification | This study |
| pRLAlr3858-CFP | Km^r^ Nm^r^; pRL277 carrying 161 to 1284 of *ftsZ* *+ cfp + kanamycin resistance cassette +* 1353 to 2375 of *ftsZ*, used for FtsZ localization | This study |
| pKT25 | Km^r^; empty vector (encoding the T25 fragment), used for BACTH assay | 4 |
| pUT18C | Car^r^; empty vector (encoding the T18 fragment), used for BACTH assay | 4 |
| pKT25-zipA | Km^r^; positive control, used for BACTH assay | 4 |
| pUT18C-zipA | Car^r^; positive control, used for BACTH assay | 4 |
| pKT25-hetF | Km^r^; pKT25 carrying *hetF* ORF, used for BACTH assay | This study |
| pUT18C-hetF | Car^r^; pUT18C carrying *hetF* ORF, used for BACTH assay | This study |
| pKT25-ftsI | Km^r^; pKT25 carrying *ftsI* ORF, used for BACTH assay | This study |
| pUT18C-ftsI | Car^r^; pUT18C carrying *ftsI* ORF, used for BACTH assay | This study |
| pKT25-ftsZ | Km^r^; pKT25 carrying *ftsZ* ORF, used for BACTH assay | This study |
| pUT18C-ftsZ | Car^r^; pUT18C carrying *ftsZ* ORF, used for BACTH assay | This study |
| pKT25-sepF | Km^r^; pKT25 carrying *sepF* ORF, used for BACTH assay | This study |
| pUT18C- sepF | Car^r^; pUT18C carrying *sepF* ORF, used for BACTH assay | This study |
| pKT25-ftsW | Km^r^; pKT25 carrying *ftsW* ORF, used for BACTH assay | This study |
| pUT18C-ftsW | Car^r^; pUT18C carrying *ftsW* ORF, used for BACTH assay | This study |
| pKT25-pbp2 | Km^r^; pKT25 carrying *pbp2* ORF, used for BACTH assay | This study |
| pUT18C-pbp2 | Car^r^; pUT18C carrying *pbp2* ORF, used for BACTH assay | This study |
| pKT25-hetF_E130R_ | Km^r^; pKT25 carrying *hetF*_E130R_ ORF, used for BACTH assay | This study |
| pUT18C-hetF_E130R_ | Car^r^; pUT18C carrying *hetF*_E130R_ ORF, used for BACTH assay | This study |
| pKT25-hetF_E130G_ | Km^r^; pKT25 carrying *hetF*E_130G_ ORF, used for BACTH assay | This study |
| pUT18C-hetF_E130G_ | Car^r^; pUT18C carrying *hetF*_E130G_ ORF, used for BACTH assay | This study |
| pKT25-hetF_E130A_ | Km^r^; pKT25 carrying *hetF*_E130A_ ORF, used for BACTH assay | This study |
| pUT18C-hetF_E130A_ | Car^r^; pUT18C carrying *hetF*_E130A_ ORF, used for BACTH assay | This study |
| pKT25-hetF_L278S_ | Km^r^; pKT25 carrying *hetF*_L278S_ ORF, used for BACTH assay | This study |
| pUT18C-hetF_L278S_ | Car^r^; pUT18C carrying *hetF*_L278S_ ORF, used for BACTH assay | This study |
| pKT25-hetF_L278A_ | Km^r^; pKT25 carrying *hetF*_L278A_ ORF, used for BACTH assay | This study |
| pUT18C-hetF_L278A_ | Car^r^; pUT18C carrying *hetF*_L278A_ ORF, used for BACTH assay | This study |

*^a^* Km, kanamycin; Nm, neomycin; Sm, streptomycin; Sp, spectinomycin; Car: carbenicillin.

**REFERENCES**

1. Niu T-C, Lin GM, Xie LR, Wang ZQ, Xing WY, Zhang JY, Zhang CC. 2018. Expanding the potential of CRISPR-Cpf1 based genome editing technology in the cyanobacterium *Anabaena* PCC 7120. ACS Synth Biol 8:170-180. https://doi.org/10.1021/acssynbio.8b00437.

2. Zhang SR, Lin GM, Chen WL, Wang L, Zhang CC. 2013. ppGpp metabolism is involved in heterocyst development in the cyanobacterium *Anabaena* sp. strain PCC 7120. J Bacteriol 195:4536–4544. https://doi.org/10.1128/JB.00724-13.

3. Xing WY, Xie LR, Zeng X, Yang Y, Zhang CC. 2020. Functional dissection of genes encoding DNA polymerases based on conditional mutants in the heterocyst-forming cyanobacterium *Anabaena* PCC 7120. Front Microbiol 11:1108. <https://doi.org/10.3389/fmicb.2020.01108>.

4. Battesti A, Bouveret E. 2012. The bacterial two-hybrid system based on adenylate cyclase reconstitution in *Escherichia coli*. Methods 58:325–334. https://doi.org/10.1016/j.ymeth.2012.07.018.
